# Comparative efficacy and safety of botanical drugs for mild cognitive impairment: a systematic review and network meta-analysis

**DOI:** 10.3389/fphar.2025.1657169

**Published:** 2025-11-17

**Authors:** Yifan Yang, Haixia Wang, Jianmeng Lü, Hui Zhang, Tao Wang, Yongmei Yan

**Affiliations:** 1 Encephalopathy Hospital, Affiliated Hospital of Shaanxi University of Chinese Medicine, Xianyang, Shaanxi, China; 2 The Second Clinical Medical School, Shaanxi University of Chinese Medicine, Xianyang, Shaanxi, China; 3 Neurology Department, Shaanxi Provincial People’s Hospital, Xi’an, Shaanxi, China

**Keywords:** plant extracts, mild cognitive impairment, cognitive function, neuroprotection, network meta-analysis and systematic review

## Abstract

**Objective:**

This study evaluated the comparative efficacy and safety of eighteen botanical drug interventions in improving cognitive function, daily living activities, and psychological wellbeing in patients with mild cognitive impairment (MCI).

**Methods:**

Randomized controlled trials were identified from PubMed, Embase, Cochrane Library, and Web of Science up to September 2025. Methodological quality was assessed using the Cochrane Risk of Bias tool, and data were analyzed within a Bayesian framework.

**Results:**

Nineteen trials involving 4,956 participants were included. For cognitive function, measured as a standardized mean difference (SMD) to harmonize various assessment scales, Pycnogenol showed the most significant effect and the highest probability of being the best intervention (Surface Under the Cumulative Ranking Curve, SUCRA: 98.8%). It also ranked highest for improving daily living (SUCRA: 100%), whereas Cosmos caudatus Supplement ranked first for psychological wellbeing (SUCRA: 98.9%). Most included botanical drugs were generally well-tolerated, with adverse event rates comparable to placebo, although safety reporting was inconsistent across studies.

**Conclusion:**

Pycnogenol showed the highest probability of improving cognitive function and daily living scores in patients with MCI, while Cosmos caudatus supplementation ranked highest for psychological outcomes. Although these findings highlight potential benefits, heterogeneity among the included studies warrant cautious interpretation.

## 1 Introduction

Mild cognitive impairment (MCI) represents a transitional state between normal aging and dementia, with increasing prevalence among older adults (Mian et al., 2024). Research indicates that approximately 10%–20% of elderly individuals aged 65 and older experience MCI (Petersen et al., 2009), with 10%–15% of these patients progressing to dementia each year (Eshkoor et al., 2015). MCI typically affects one or more cognitive domains, which not only disrupts daily life (Winblad et al., 2004) but also frequently leads to significant psychological distress, including symptoms of depression and a decline in overall quality of life (Dietlin et al., 2019). Therefore, timely intervention is crucial to mitigate the onset and progression of dementia, ultimately improving patients’ quality of life.

Currently, treatment options for MCI remain limited and often fail to prevent progression to dementia, highlighting the need for alternative therapies (Anderson and Malone, 2022). Therefore, plant-derived metabolites have been proposed as neuroprotective agents due to their antioxidant and anti-inflammatory properties. Plant extracts are rich in bioactive metabolites, including flavonoids, polyphenols, and alkaloids. These metabolites have been shown to improve nerve cell function and protect neurons from damage (Liu et al., 2023; Rajaram et al., 2019). Clinical research indicates that plant extracts enhance memory, cognitive abilities, and improve symptoms of neurodegenerative diseases, as demonstrated by the active metabolites found in plants such as Ginkgo biloba leaves, ginseng, and kudzu root. Significant progress has been made in mitigating or delaying the progression of cognitive decline (Kandiah et al., 2021; Lee et al., 2024; Wang et al., 2022). Previous research suggests that its mechanisms of action include promoting cognitive function through antioxidative and anti-inflammatory pathways, modulating neurotransmitters, and enhancing neuronal growth and connectivity (Ren and Qu, 2023; Yao et al., 2021; Zhao et al., 2021). Thus, plant extracts show promising potential in the management of mild cognitive impairment. However, their relative efficacy remains uncertain. This study aimed to synthesize evidence from randomized controlled trials through a network meta-analysis (NMA) to rank the relative effects of plant extracts in improving cognitive and functional outcomes in patients with MCI. The term ‘effects’ is deliberately used here to accurately reflect the synthesis of outcomes from multiple, heterogeneous trials, whereas “efficacy” typically implies performance under ideal, controlled conditions.

## 2 Materials and methods

This systematic review and network meta-analysis was conducted and reported in accordance with the Preferred Reporting Items for Systematic Reviews and Meta-Analyses (PRISMA) 2020 statement. The completed PRISMA checklist is available in Supplementary Table S7.

### 2.1 Search strategy

This systematic review and network meta-analysis was conducted following a prospectively registered protocol (PROSPERO CRD42022324641), designed to address a specific clinical question structured around the PICOTS (Population, Intervention, Comparator, Outcome, Timing, and Setting) framework. Five electronic databases were searched (PubMed, EMBASE, Cochrane Central Register of Controlled Trials, and Web of Science) for studies conducted from creation to September 2025, using the PICOS search strategy of (P) Population: people with mild cognitive impairment; (I) Intervention: plant extracts; (C) Comparison: control group with placebo; (O) Outcomes: people with mild cognitive impairment, cognitive testing; (S) Study type: randomized controlled trials (RCTs). Studies were retrieved from the search strategy and manual searches. The complete search strategies used for all searched databases are provided in Supplementary Table S1. To ensure a comprehensive and unbiased retrieval of literature, our search strategy deliberately employed broad terms for the intervention metabolite (e.g., “Plant Extracts”, “botanical drugal Medicines”) rather than enumerating specific plant names. This approach was chosen to maximize breadth initially and avoid the potential omission of relevant studies on less common or newly investigated extracts. The sensitivity search was followed by a meticulous two-stage manual screening process to minimize the risk of missing eligible studies. Furthermore, we supplemented our electronic search with a manual review of reference lists from included articles and relevant reviews, a process that successfully identified several additional studies for inclusion.

### 2.2 Inclusion criteria

(1) Patients meeting the criteria of Mild Cognitive Impairment (MCI) according to the diagnostic criteria of National Institute on Aging - Alzheimer’s Association (NIA - AA) and Peterson criteria; (2) Various plant extracts are used as intervention in the experimental group; (3) Placebo treatment or normal care is used in the control group; (4) Clinical randomized controlled; (5) The outcome measures of the included trials had to assess at least one of the following three core domains: (a) Cognitive Function, measured by validated scales (e.g., MMSE, MoCA); (b) Activities of Daily Living; and (c) Psychological Wellbeing, including assessments for mood (e.g., Geriatric Depression Scale) or quality of life. This three-pronged approach was chosen to capture the multifaceted impact of interventions on MCI.

### 2.3 Exclusion criteria

(1) Studies with incomplete or missing data; 2) Non-randomized controlled trials, quasi-randomized trials, animal studies, protocols, conference abstracts, case reports, and correspondence; 3) Control groups not meeting the requirements of proper controls; 4) Participants with any type of dementia, mild dementia; 5) Botanical therapies, acupuncture, physical exercise, granule formulations.

### 2.4 Study selection

Literature was excluded using EndNote20 to manage literature. Two researchers screened the title to exclude duplicates, non-randomized controlled trials, review papers, conference papers, protocols, and correspondence. Two researchers reviewed abstracts to decide to include or exclude. Finally, the remaining literature was fully read by the researchers and evaluated as to whether to be selected into the final literature. The researchers independently screened the literature and compared them. If the two researchers agreed with each other, the literature was selected. If not, it could be discussed and decided by the third researcher.

### 2.5 Data extraction

A seven - item data extraction table was developed to extract information as follows: author, year of publication, country, period, sample size, mean age, information about the plant extract intervention.

### 2.6 Risk of bias in individual studies

The bias risk (i.e., risk of bias) of RCTs was evaluated using Cochrane Handbook version 5.1.0 tool. Seven sources were examined including (1) randomized sequence generation, (2) treatment allocation concealment, (3) blinding of participants, (4) blinding of personnel, (5) incomplete outcome data, (6) selective reporting and (7) other. All trials were classified into three ROB categories based on the number of domains (high, moderate, and low) for which ROB was considered high (five or more domains), moderate (three or four domains), or low (two or fewer domains) (Higgins et al., 2011). The ROB was then rated independently by two reviewers to decide the eligibility, with disagreements being resolved by a third reviewer.

### 2.7 Data analysis

For all continuous outcomes, data were presented as means with standard deviation (Zeng et al., 2022). For outcomes assessed using different scales, such as cognitive function (e.g., MMSE, MoCA, ADAS-cog), the standardized mean difference (SMD) with Hedges’ g correction for small sample bias was used as the summary effect measure. This approach harmonizes results from various instruments onto a single, comparable scale. Prior to pooling, the direction of outcomes was standardized so that a positive SMD consistently represented an improvement. For outcomes measured with a single, consistent scale across trials, the mean difference (MD) was used. Both measures will be presented with 95% confidence intervals (CI). Due to the potential heterogeneity of studies, we opted for a random effects rather than a fixed effects analysis random (Jackson, Riley and White, 2011).

We employed Stata software (Stata, 15.1) and the Markov chain Monte Carlo simulation in Bayesian model with the principle for the guidelines for the reports of Systematic reviews (PRISMA) NMA (Harrington et al., 2021; Shamseer et al., 2015). to quantify and demonstrate the consistency between the indirect comparisons and the direct comparison using the nodal method (Stata software. P-value >0.05 means consistency test passing (Salanti et al., 2011). Assessment of Transitivity and Consistency Assumptions. The validity of a network meta-analysis hinges on the assumptions of transitivity and consistency.

Transitivity is the assumption that indirect evidence is a valid substitute for direct evidence. We assessed this conceptually by examining the distribution of potential clinical and methodological effect modifiers across the different pairwise comparisons. These modifiers included patient age, baseline cognitive scores, and study duration, as summarized in Table 2. We judged the transitivity assumption to be plausible if these characteristics were broadly similar across studies comparing different interventions to a common comparator (i.e., placebo).

Consistency refers to the agreement between direct and indirect evidence. We statistically evaluated consistency for each closed loop in the network using the node-splitting method within our Bayesian framework. This method separates the evidence for a specific comparison (node) into direct and indirect metabolites and calculates the difference between them, along with a Bayesian P-value. A P-value >0.05 was considered to indicate no significant inconsistency between the direct and indirect evidence. Any significant deviations would be explicitly reported and investigated. The detailed statistical report, including our Stata scripts, analysis logs, and the full outputs from the inconsistency tests, is provided in Supplementary File *S1.*

Stata software was utilized to generate and characterize the network diagrams of plant extracts. In the network diagram of plant extract intervention, each node corresponds to a plant extract intervention or a control state, while the lines between nodes depict the direct head - to - head comparison of interventions. 2010). The width of the line and the size of the nodes are also correlated with the number of studies included (Chaimani et al., 2013).

The intervention hierarchy was summarised and reported with a P score. The P score is regarded as a frequentist equivalent to the SUCRA values and indicates the degree of evidence for a treatment being better than another treatment, averaged over all other treatments. The P score varies from 0 to 1, where the best treatment with no uncertainty of treatment effect is 1, and the worst treatment with no uncertainty is 0. While P score or SUCRA can be further re-expressed as the percentage of effectiveness or acceptability of the plant extract interventions, scores should be interpreted cautiously, unless there were real clinically meaningful differences between interventions (H. Liu et al., 2024). A network funnel plot was created and visually assessed based on the criterion of symmetry to detect bias from small-scale studies which can lead to publication bias in NMA (Shi et al., 2024).

## 3 Results

### 3.1 Study and identification and selection

**FIGURE 1:**
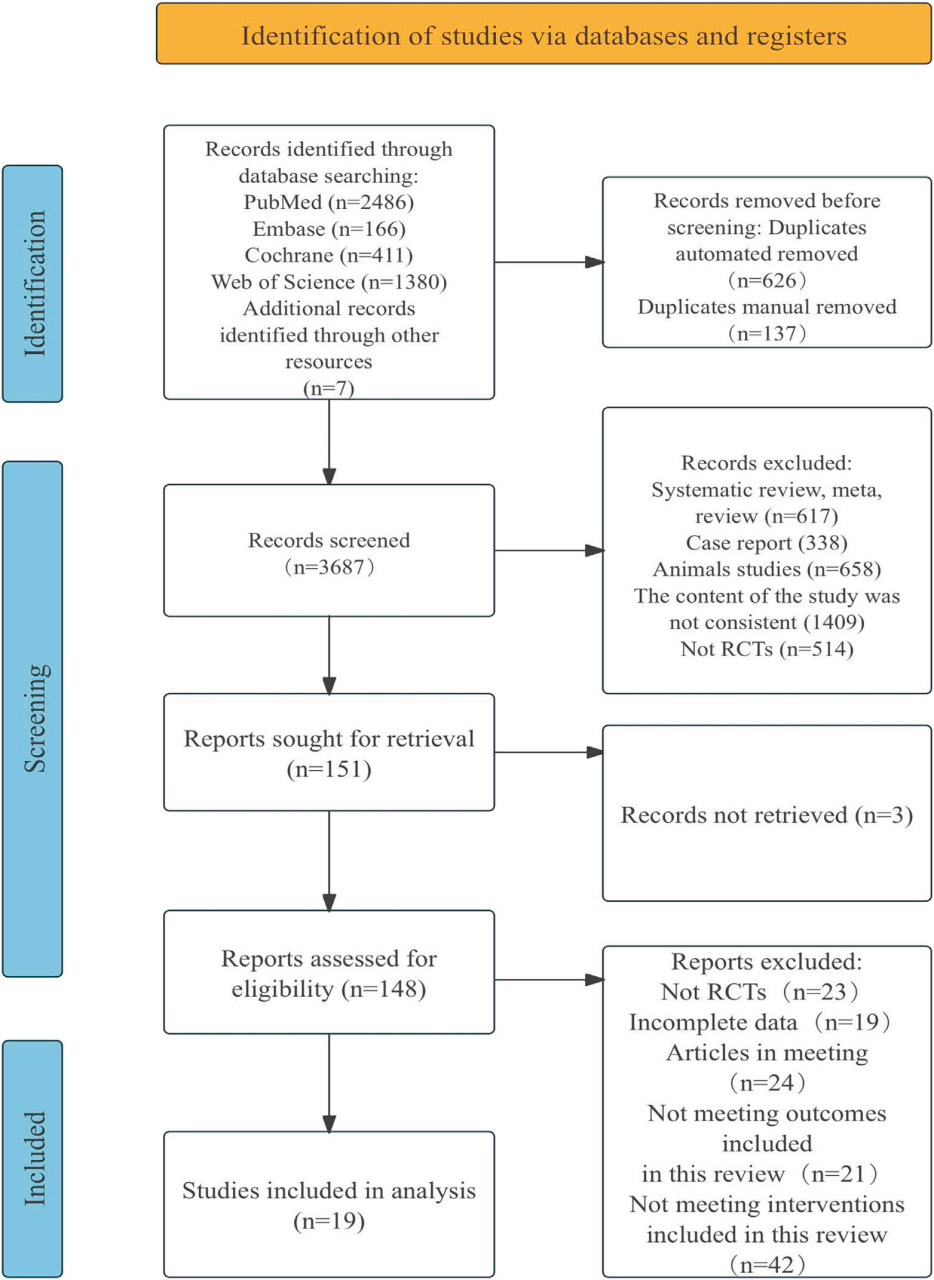
Flow diagram of literature selection.

A total of 4,450 documents were retrieved from electronic databases, with an additional seven documents obtained through manual searches. After removing duplicates, 3,687 documents were screened by title and abstract, resulting in the exclusion of 3,536 documents. The remaining 151 documents were reviewed in full, and 132 were excluded for reasons such as non-randomized controlled trials, having incomplete data, being conference papers, or failing to meet the intervention criteria for this review. This has 19 documents to be included in the study. Flow diagram of literature selection in Figure 1.

### 3.2 Quality assessment of the included studies

**FIGURE 2:**
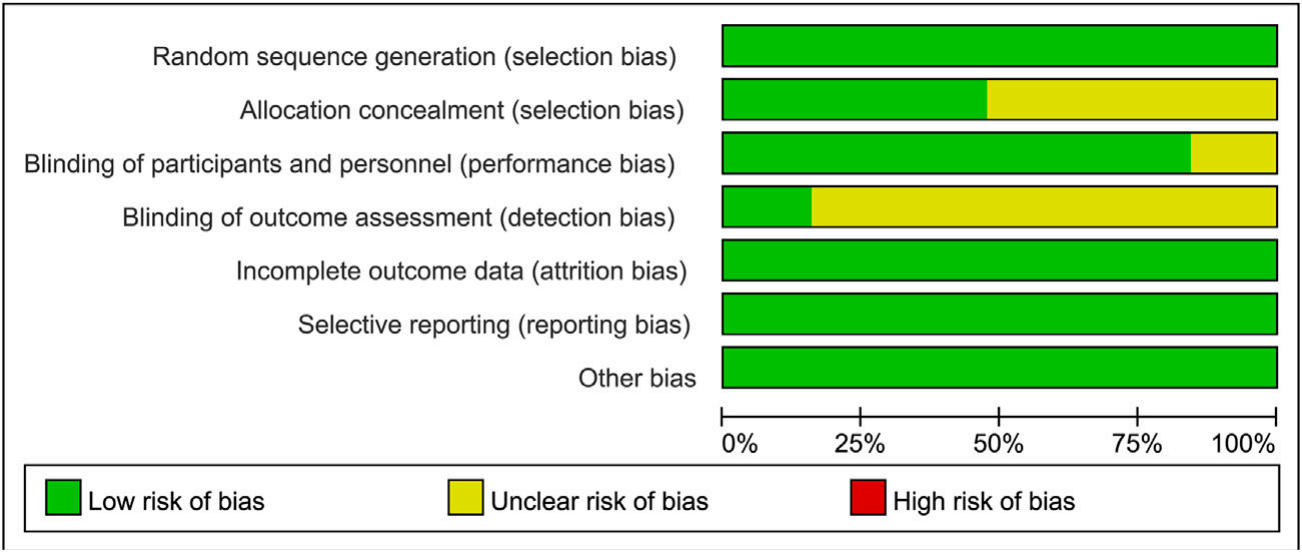
Risk of bias graph for all included studies.

**FIGURE 3:**
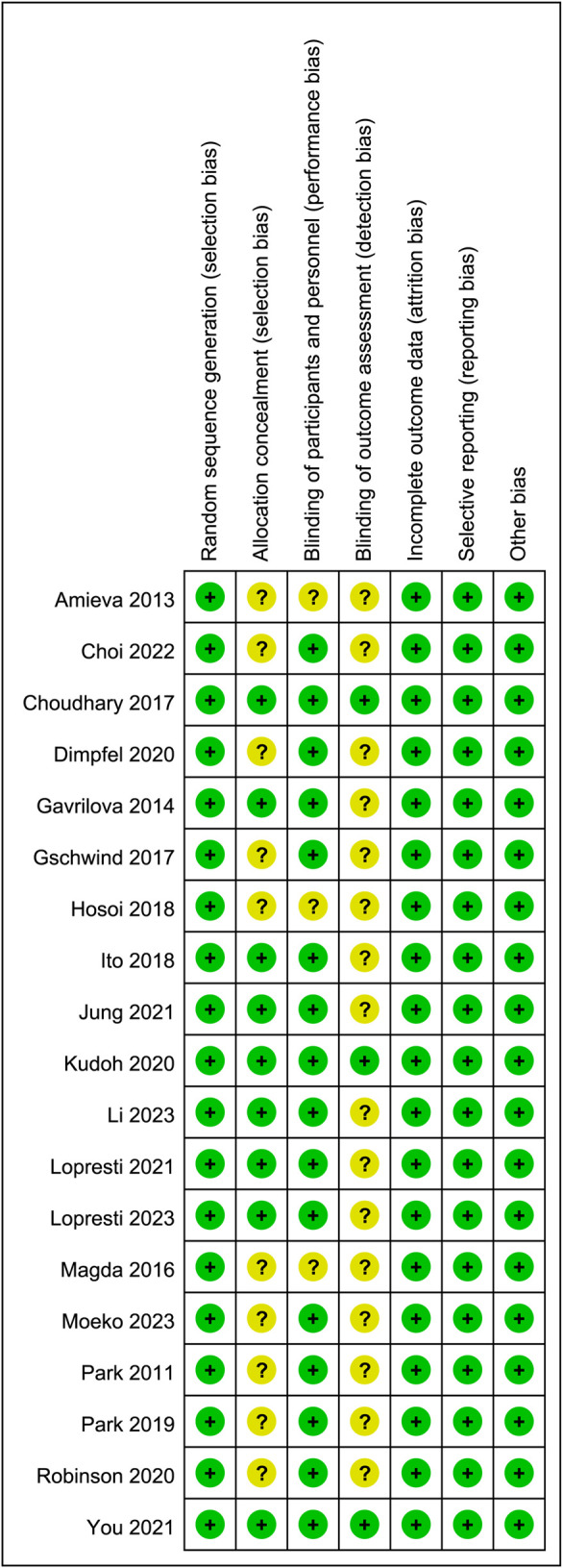
Risk of bias summary for all included studies.

Sixteen studies were identified as low-risk, and 3 as medium-risk. The risk of bias assessment indicated that 16 RCTs specified the methods used for random sequence generation; 9 RCTs provided detailed descriptions of the allocation concealment methods used; and 3 RCTs outlined the blinding techniques employed for outcome assessment. While most domains showed low risk, several studies exhibited unclear or high risk in allocation concealment and blinding of participants and personnel, as shown in Figures 2, 3. A detailed breakdown of the risk of bias assessment for each individual study is available in Supplementary Figure S2. All included studies reported the loss to follow-up, demonstrating a high level of data completeness.

### 3.3 Characteristics of the included studies

In total, 19 randomized controlled trials involving 4,956 patients diagnosed with mild cognitive impairment were included in this network meta-analysis. These studies evaluated the effects of 18 distinct plant extract interventions against a placebo control.

The specific interventions analyzed were: Ginkgo biloba extract (EGb761), SM70EE (from Morus alba), Ashwagandha (Withania somnifera), AdaptraForte (a proprietary blend), LI1370 (a proprietary Bacopa monnieri extract), Pycnogenol® (from *Pinus pinaster*), SOCE (Sophorae Fructus and Olibanum complex), Sabroxy® (from Oroxylum indicum), Memophenol™ (a grape and blueberry blend), Mofficinalis (Melissa officinalis), Ginseng (Panax ginseng), LGNC07 (a complex botanical drugal formula), CCE (Cistanche tubulosa), Crocus (Crocus sativus), Cosmos Caudatus Supplement (CCSupplement), AS (Angelica archangelica), GSPE (Grape Seed Proanthocyanidin Extract), and Feruguard. A detailed description of each preparation, including its botanical taxonomy, plant part used, and standardization details (as reported in the original studies), is provided in Supplementary Table S2.

Regarding the outcomes assessed, nineteen studies reported on cognitive function, five on daily living, and four on psychological wellbeing. The geographical distribution of the studies was diverse, with eight conducted in East Asia, one in the Americas, sixteen in Europe, two in Oceania, one in South Asia, and one in Southeast Asia. The detailed characteristics of each included study are presented in Table 1.

**TABLE 1:** Characteristics of the studies included in the meta-analysis.

Author	Country	Year	Population	Age (mean + SD)	Total/male/female	Intervention	Control	Dosage/Regimen	Duration	Outcome
Amieva	France	2013	MCI	T:74.8 (6.6) C:75.0 (6.9)	T:589/154/435 C:2874/1318/1556	EGb761	Placebo	NA	20-year	MMSE
Choi	Korea	2022	MCI	T:67.68 (4.43) C:68.85 (4.89)	T:40/12/28 C:40/9/31	SM70EE	Placebo	1 g/day,thrice daily	12 weeks	MoCA
Choudhary	India	2017	MCI	C:50 (7.33) C:51 (7.98)	T:25/NA/NAC:25/NA/NA	Ashwagandha	Placebo t	300 mg/time, twice daily	8 weeks	Wisconsin card sort test
Dimpfel	Germany	2020	MCI	T:63.81 (2.04) C:63.81 (2.04)	T:16/8/8 C:16/8/8	AdaptraForte	Placebo	NA	4 weeks	d2 Test
										SF-B/R
Gschwind	Switzerland	2017	MCI	T:67.8 (8.3) C:69.2 (8.6)	T:25/10/15 C:25/15/10	LI1370	Placebo	120 mg/time, twice daily	6 months	Working memory dual-task Habitual walking
Hosoi	Italy	2018	MCI	T:NA C:NA	T:43/43/0 C:44/44/0	Pycnogenol	Placebo	150 mg/day	8 weeks	MMSE
										Simple daily tasks Visual Analogue Scale
Jung	Korea	2021	MCI	T:68.69 (5.33) C:71.14 (5.62)	T:35/9/26 C:35/16/19	SOCE	Placebo	1.5 g/day,thrice daily	12 weeks	Computerized neurocognitive function test
Lopresti	Australia	2021	MCI	T:66.07 (0.73) C:68.28 (0.9)	T:42/9/33 C:40/12/28	Sabroxy	Placebo	500 mg/time, twice daily	12 weeks	MoCA CASP-19
Lopresti	Australia	2023	MCI	T:68.36 (0.54) C:67.37 (0.62)	T:73/19/54 C:70/20/50	Memophenol	Placebo	300 mg/day,twice daily	24 weeks	CFQ CASP-19
Moeko	Japan	2023	MCI	T:71.55 (4.14) C:71.65 (4.21)	T:162/56/106 C:161/57/104	Mofficinalis	Placebo	500 mg/day	96 weeks	MMSE
Park	Korea	2019	MCI	T:61.8 (6.9) C:62.6 (6.3)	T:45/15/30 C:45/15/30	Ginseng	Placebo	3g/day	24 weeks	MMSE
										K-IADL
Park	Korea	2011	MCI	T:57.58 (9.45) C:56.28 (9.92)	T:45/13/32 C:46/12/34	LGNC07	Placebo	860 mg/time, twice daily	16 weeks	Rey–Kim memory test
Robinson	Mexico	2020	MCI	T:59.53 (3.31) C:59.06 (3.65)	T:19/8/11 C:18/8/10	CCE	Placebo	100 mg/time, twice daily	4 weeks	N-back
Magda	Greece	2016	MCI	T:71.47 (6.73) C:69.72 (7.33)	T:17/5/12 C:18/4/14	Crocus	Placebo	NA	12 months	MMSE
										GDS
You	Malaysia	2021	MCI	T:65.83 (4.35) C:64.42 (3.71)	T:24/8/16 C:24/8/16	Cosmos Caudatus Supplement (CCSupplement)	Placebo	500 mg/day	12 weeks	MMSE
										POMS
Gavrilova	Germany	2014	MCI	T:65 (7) C:63 (7)	T:80/22/58 C:79/13/66	EGb761	Placebo	240 mg/day	24 weeks	TMT
										NPI
Kudoh	Japan	2020	MCI	T:NA C:NA	T:24/11/13 C:23/8/15	Feruguard	Placebo	240 mg/day,twice daily	24 weeks	MMSE
Ito	Japan	2018	MCI	T:70.4 (7.0) C:68.4 (7.3)	T:7/5/2 C:7/4/3	AS	Placebo	10 mg/day	12 weeks	ADAS-cog
Li	China	2023	MCI	T:68.91 (4.91) C:70.53 (5.34)	T:35/24/11 C:36/19/17	GSPE	Placebo	320 mg/day	6 months	MoCA

T, experimental group; C, control group; MMSE:Mini-Mental State Examination; MoCA, montreal cognitive assessment; ADAS-cog, Alzheimer’s Disease Assessment Scale-Cognitive Subscale; SF-B/R, sleep questionnaire; CASP-19, Quality of life for the elderly-19; CFQ, cognitive fusion questionnaire; K-IADL, Korea-Instrumental Activities of Daily Living; GDS, geriatric depression scale; POMS, Profile of Mood States; TMT, tinetti mobility test; NPI, neuropsychiatric inventory; NA, unavailable.

### 3.4 Network meta-analysis

Before presenting the results of the synthesis, the core assumptions of the network meta-analysis were evaluated. The assumption of transitivity was deemed acceptable, as the baseline characteristics of participants and key study design features (as shown in Table 2) were judged to be sufficiently similar across the different sets of trials that informed the indirect comparisons. The statistical assessment of consistency using the node-splitting method revealed no significant inconsistencies between direct and indirect evidence for any of the outcome networks (all Bayesian *P-*values >0.05). The detailed outputs of these tests are available in Supplementary File *S*1. Therefore, the data were considered suitable for network meta-analysis.

**TABLE 2:** League table of standardized mean differences (SMD) on cognitive function.

Pycnogenol	CCSupplement	Feruguard	Ashwagandha	AdaptraForte	SOCE	AS	CCE	Crocus	LI1370	SM70EE	Ginseng	Mofficinalis	GSPE	Placebo	LGNC07	EGb761	Sabroxy	Memophenol
Pycnogenol	--0.07 (−0.32, 0.18)	−0.15 (−0.41, 0.11)	−0.98 (−1.75, −0.21)	−1.04 (−1.94, −0.13)	−1.22 (−1.98, −0.47)	−1.25 (−2.44, −0.05)	−1.34 (−2.19, −0.49)	−1.39 (−2.23, −0.55)	−1.44 (−2.24, −0.64)	−1.62 (−2.30, −0.93)	−1.73 (−2.39, −1.06)	−1.74 (−2.30, −1.18)	−1.75 (−2.44, −1.06)	−0.85 (−1.05, −0.65)	−1.93 (−2.58, −1.28)	−1.90 (−2.41, −1.38)	−2.23 (−2.91, −1.54)	−2.33 (−2.95, −1.70)
0.07 (−0.18, 0.32)	CCSupplement	−0.08 (−0.32, 0.16)	−0.45 (−1.31,0.42)	−0.50 (−1.49,0.48)	−0.69 (−1.54,0.16)	−0.71 (−1.97,0.54)	−0.81 (−1.75,0.13)	−0.86 (−1.78,0.07)	−0.91 (−1.80, −0.02)	−1.09 (−1.87, −0.30)	−1.19 (−1.96, −0.42)	−1.21 (−1.89, −0.53)	−1.21 (−2.00, −0.42)	−0.78 (−1.04, −0.52)	−1.40 (−2.16, −0.64)	−1.37 (−2.01, −0.72)	−1.69 (−2.48, −0.90)	−1.79 (−2.53, −1.06)
0.15 (−0.11, 0.41)	0.08 (−0.16, 0.32)	Feruguard	−0.16 (−1.01,0.69)	−0.21 (−1.18,0.76)	−0.40 (−1.23,0.43)	−0.42 (−1.67,0.82)	−0.52 (−1.44,0.40)	−0.57 (−1.48,0.34)	−0.62 (−1.49,0.25)	−0.80 (−1.56, −0.03)	−0.90 (−1.66, −0.15)	−0.92 (−1.58, −0.26)	−0.93 (−1.70, −0.16)	−0.70 (−0.95, −0.45)	−1.11 (−1.85, −0.37)	−1.08 (−1.70, −0.46)	−1.40 (−2.17, −0.64)	−1.51 (−2.22, −0.79)
0.98 (0.21,1.75)	0.45 (−0.42,1.31)	0.16 (−0.69,1.01)	Ashwagandha	−0.05 (−1.01,0.90)	−0.24 (−1.05,0.57)	−0.27 (−1.49,0.96)	−0.36 (−1.26,0.54)	−0.41 (−1.30,0.48)	−0.46 (−1.31,0.39)	−0.64 (−1.38,0.10)	−0.75 (−1.47, −0.02)	−0.76 (−1.39, −0.13)	−0.77 (−1.51, −0.02)	−0.65 (−0.89, −0.41)	−0.95 (−1.66, −0.23)	−0.92 (−1.51, −0.33)	−1.25 (−1.99, −0.50)	−1.35 (−2.03, −0.66)
1.04 (0.13,1.94)	0.50 (−0.48,1.49)	0.21 (−0.76,1.18)	0.05 (−0.90,1.01)	AdaptraForte	−0.19 (−1.13,0.75)	−0.21 (−1.53,1.11)	−0.31 (−1.32,0.71)	−0.35 (−1.36,0.66)	−0.40 (−1.38,0.57)	−0.58 (−1.46,0.30)	−0.69 (−1.56,0.18)	−0.70 (−1.49,0.08)	−0.71 (−1.59,0.17)	−0.61 (−0.87, −0.35)	−0.89 (−1.75, −0.04)	−0.86 (−1.62, −0.11)	−1.19 (−2.07, −0.31)	−1.29 (−2.13, −0.46)
1.22 (0.47,1.98)	0.69 (−0.16,1.54)	0.40 (−0.43,1.23)	0.24 (−0.57,1.05)	0.19 (−0.75,1.13)	SOCE	−0.02 (−1.24,1.20)	−0.12 (−1.00,0.77)	−0.16 (−1.04,0.71)	−0.22 (−1.05,0.62)	−0.39 (−1.12,0.33)	−0.50 (−1.21,0.21)	−0.52 (−1.13,0.09)	−0.52 (−1.25,0.21)	−0.64 (−1.20, −0.08)	−0.71 (−1.40, −0.01)	−0.67 (−1.24, −0.11)	−1.00 (−1.73, −0.27)	−1.10 (−1.77, −0.43)
1.25 (0.05,2.44)	0.71 (−0.54,1.97)	0.42 (−0.82,1.67)	0.27 (−0.96,1.49)	0.21 (−1.11,1.53)	0.02 (−1.20,1.24)	AS	−0.09 (−1.37,1.19)	−0.14 (−1.42,1.13)	−0.19 (−1.44,1.05)	−0.37 (−1.55,0.80)	−0.48 (−1.64,0.68)	−0.49 (−1.60,0.61)	−0.50 (−1.68,0.68)	−0.62 (−1.70,0.46)	−0.68 (−1.84,0.47)	−0.65 (−1.74,0.43)	−0.98 (−2.16,0.20)	−1.08 (−2.22,0.06)
1.34 (0.49,2.19)	0.81 (−0.13,1.75)	0.52 (−0.40,1.44)	0.36 (−0.54,1.26)	0.31 (−0.71,1.32)	0.12 (−0.77,1.00)	0.09 (−1.19,1.37)	CCE	−0.05 (−1.01,0.91)	−0.10 (−1.02,0.83)	−0.28 (−1.10,0.55)	−0.39 (−1.20,0.43)	−0.40 (−1.13,0.33)	−0.41 (−1.24,0.42)	−0.52 (−1.21,0.17)	−0.59 (−1.39,0.21)	−0.56 (−1.25,0.13)	−0.89 (−1.71, −0.06)	−0.99 (−1.76, −0.21)
1.39 (0.55,2.23)	0.86 (−0.07,1.78)	0.57 (−0.34,1.48)	0.41 (−0.48,1.30)	0.35 (−0.66,1.36)	0.16 (−0.71,1.04)	0.14 (−1.13,1.42)	0.05 (−0.91,1.01)	Crocus	−0.05 (−0.97,0.86)	−0.23 (−1.04,0.58)	−0.34 (−1.14,0.46)	−0.35 (−1.07,0.36)	−0.36 (−1.18,0.46)	−0.47 (−1.15,0.20)	−0.54 (−1.33,0.25)	−0.51 (−1.19,0.17)	−0.84 (−1.66, −0.02)	−0.94 (−1.70, −0.17)
1.44 (0.64,2.24)	0.91 (0.02,1.80)	0.62 (−0.25,1.49)	0.46 (−0.39,1.31)	0.40 (−0.57,1.38)	0.22 (−0.62,1.05)	0.19 (−1.05,1.44)	0.10 (−0.83,1.02)	0.05 (−0.86,0.97)	LI1370	−0.18 (−0.95,0.59)	−0.29 (−1.04,0.47)	−0.30 (−0.96,0.36)	−0.31 (−1.08,0.47)	−0.42 (−1.04,0.20)	−0.49 (−1.23,0.25)	−0.46 (−1.08,0.17)	−0.79 (−1.56, −0.01)	−0.89 (−1.61, −0.17)
1.62 (0.93,2.30)	1.09 (0.30,1.87)	0.80 (0.03,1.56)	0.64 (−0.10,1.38)	0.58 (−0.30,1.46)	0.39 (−0.33,1.12)	0.37 (−0.80,1.55)	0.28 (−0.55,1.10)	0.23 (−0.58,1.04)	0.18 (−0.59,0.95)	SM70EE	−0.11 (−0.74,0.52)	−0.12 (−0.64,0.39)	−0.13 (−0.78,0.52)	−0.24 (−0.70,0.21)	−0.31 (−0.93,0.30)	−0.28 (−0.75,0.19)	−0.61 (−1.26,0.04)	−0.71 (−1.29, −0.12)
1.73 (1.06,2.39)	1.19 (0.42,1.96)	0.90 (0.15,1.66)	0.75 (0.02,1.47)	0.69 (−0.18,1.56)	0.50 (−0.21,1.21)	0.48 (−0.68,1.64)	0.39 (−0.43,1.20)	0.34 (−0.46,1.14)	0.29 (−0.47,1.04)	0.11 (−0.52,0.74)	Ginseng	−0.01 (−0.51,0.48)	−0.02 (−0.66,0.62)	−0.14 (−0.57,0.30)	−0.20 (−0.80,0.39)	−0.17 (−0.61,0.27)	−0.50 (−1.13,0.13)	−0.60 (−1.17, −0.04)
1.74 (1.18,2.30)	1.21 (0.53,1.89)	0.92 (0.26,1.58)	0.76 (0.13,1.39)	0.70 (−0.08,1.49)	0.52 (−0.09,1.13)	0.49 (−0.61,1.60)	0.40 (−0.33,1.13)	0.35 (−0.36,1.07)	0.30 (−0.36,0.96)	0.12 (−0.39,0.64)	0.01 (−0.48,0.51)	Mofficinalis	−0.01 (−0.53,0.52)	−0.12 (−0.36,0.11)	−0.19 (−0.66,0.29)	−0.16 (−0.41,0.09)	−0.49 (−1.01,0.03)	−0.59 (−1.02, −0.15)
1.75 (1.06,2.44)	1.21 (0.42,2.00)	0.93 (0.16,1.70)	0.77 (0.02,1.51)	0.71 (−0.17,1.59)	0.52 (−0.21,1.25)	0.50 (−0.68,1.68)	0.41 (−0.42,1.24)	0.36 (−0.46,1.18)	0.31 (−0.47,1.08)	0.13 (−0.52,0.78)	0.02 (−0.62,0.66)	0.01 (−0.52,0.53)	GSPE	−0.12 (−0.58,0.35)	−0.18 (−0.80,0.44)	−0.15 (−0.62,0.32)	−0.48 (−1.14,0.18)	−0.58 (−1.17,0.01)
0.85 (0.65, 1.05)1.86 (1.35,2.37)	0.78 (0.52, 1.04)	0.70 (0.45, 0.95)	0.65 (0.41, 0.89)	0.61 (0.35, 0.87)	0.64 (0.08,1.20)	0.62 (−0.46,1.70)	0.52 (−0.17,1.21)	0.47 (−0.20,1.15)	0.42 (−0.20,1.04)	0.24 (−0.21,0.70)	0.14 (−0.30,0.57)	0.12 (−0.11,0.36)	0.12 (−0.35,0.58)	Placebo	−0.07 (−0.48,0.34)	−0.04 (−0.12,0.05)	−0.36 (−0.83,0.10)	−0.15 (−0.45, −0.15)
1.93 (1.28,2.58)	1.40 (0.64,2.16)	1.11 (0.37,1.85)	0.95 (0.23,1.66)	0.89 (0.04,1.75)	0.71 (0.01,1.40)	0.68 (−0.47,1.84)	0.59 (−0.21,1.39)	0.54 (−0.25,1.33)	0.49 (−0.25,1.23)	0.31 (−0.30,0.93)	0.20 (−0.39,0.80)	0.19 (−0.29,0.66)	0.18 (−0.44,0.80)	0.07 (−0.34,0.48)	LGNC07	0.03 (−0.39,0.45)	−0.30 (−0.92,0.32)	−0.40 (−0.95,0.15)
1.90 (1.38,2.41)	1.37 (0.72,2.01)	1.08 (0.46,1.70)	0.92 (0.33,1.51)	0.86 (0.11,1.62)	0.67 (0.11,1.24)	0.65 (−0.43,1.74)	0.56 (−0.13,1.25)	0.51 (−0.17,1.19)	0.46 (−0.17,1.08)	0.28 (−0.19,0.75)	0.17 (−0.27,0.61)	0.16 (−0.09,0.41)	0.15 (−0.32,0.62)	0.04 (−0.05,0.12)	−0.03 (−0.45,0.39)	EGb761	−0.33 (−0.80,0.14)	−0.43 (−0.80, −0.06)
2.23 (1.54,2.91)	1.69 (0.90,2.48)	1.40 (0.64,2.17)	1.25 (0.50,1.99)	1.19 (0.31,2.07)	1.00 (0.27,1.73)	0.98 (−0.20,2.16)	0.89 (0.06,1.71)	0.84 (0.02,1.66)	0.79 (0.01,1.56)	0.61 (−0.04,1.26)	0.50 (−0.13,1.13)	0.49 (−0.03,1.01)	0.48 (−0.18,1.14)	0.36 (−0.10,0.83)	0.30 (−0.32,0.92)	0.33 (−0.14,0.80)	Sabroxy	−0.10 (−0.69,0.49)
2.33 (1.70,2.95)	1.79 (1.06,2.53)	1.51 (0.79,2.22)	1.35 (0.66,2.03)	1.29 (0.46,2.13)	1.10 (0.43,1.77)	1.08 (−0.06,2.22)	0.99 (0.21,1.76)	0.94 (0.17,1.70)	0.89 (0.17,1.61)	0.71 (0.12,1.29)	0.60 (0.04,1.17)	0.59 (0.15,1.02)	0.58 (−0.01,1.17)	0.15 (0.15, 0.45)	0.40 (−0.15,0.95)	0.43 (0.06,0.80)	0.10 (−0.49,0.69)	Memophenol

**FIGURE 4:**
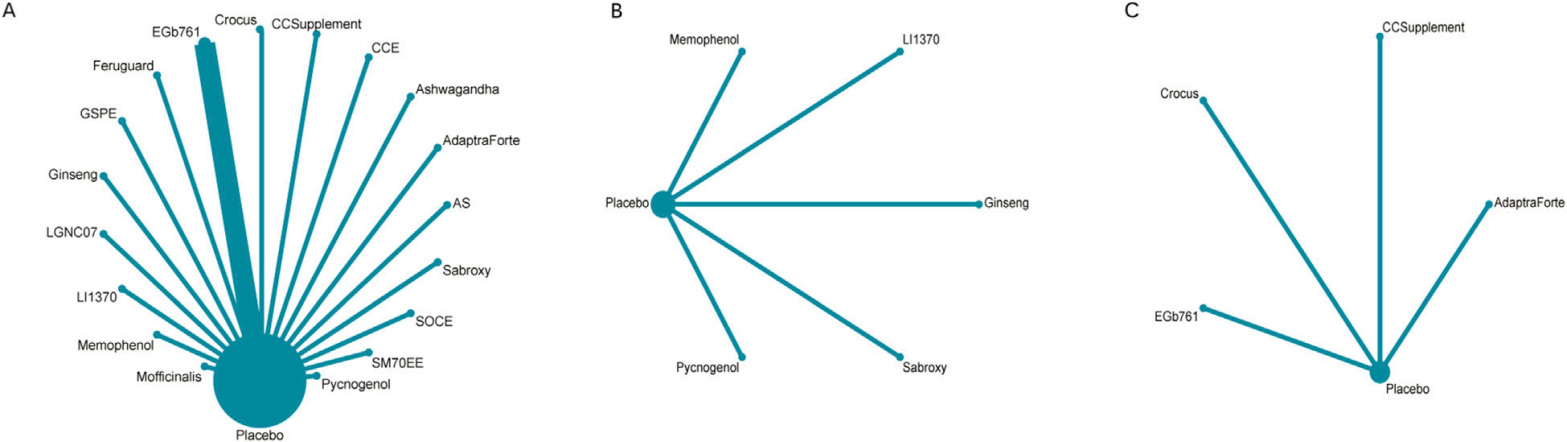
**(A)** NMA figure for cognitive function, **(B)** NMA figure for daily living, **(C)** NMA figure for psychological wellbeing.

The full NMA figure will be shown in Figures 4A–C.

Figure 4A illustrates the network of evidence for cognitive function, which included 19 interventions (18 plant extracts and placebo). Each node represents an intervention, with the size of the node proportional to the total number of participants randomized to that treatment. The lines connecting the nodes represent direct head-to-head comparisons from the included trials, and the thickness of the lines corresponds to the number of studies for each comparison. The network displays a star-shaped geometry, with placebo serving as the central and most common comparator, connected to all other active interventions. Direct comparisons between different active plant extracts were limited.

For the outcome of daily living (Figure 4B), the network was smaller, comprising six interventions. The structure was also star-shaped, with placebo as the central comparator. Pycnogenol was the intervention with the largest sample size for this outcome, as indicated by its node size.

The network for psychological wellbeing (Figure 4C) was the most sparse, connecting five interventions. This network also followed a star-shaped pattern with placebo as the common comparator, indicating that all evidence was derived from placebo-controlled trials with no direct comparisons between the active interventions themselves for this outcome.

#### 3.4.1 Cognitive function in patients with mild cognitive impairment

All P-values for indirect and direct comparisons across studies were tested for consistency and inconsistency, and the majority of P-values were greater than 0.05, indicating an acceptable level of consistency across studies. Details are provided in Supplementary Figure S1. Table 2 presents the key pairwise comparisons from the league table. The complete league table showing all comparisons is provided in Supplementary Table S4.

**FIGURE 5:**
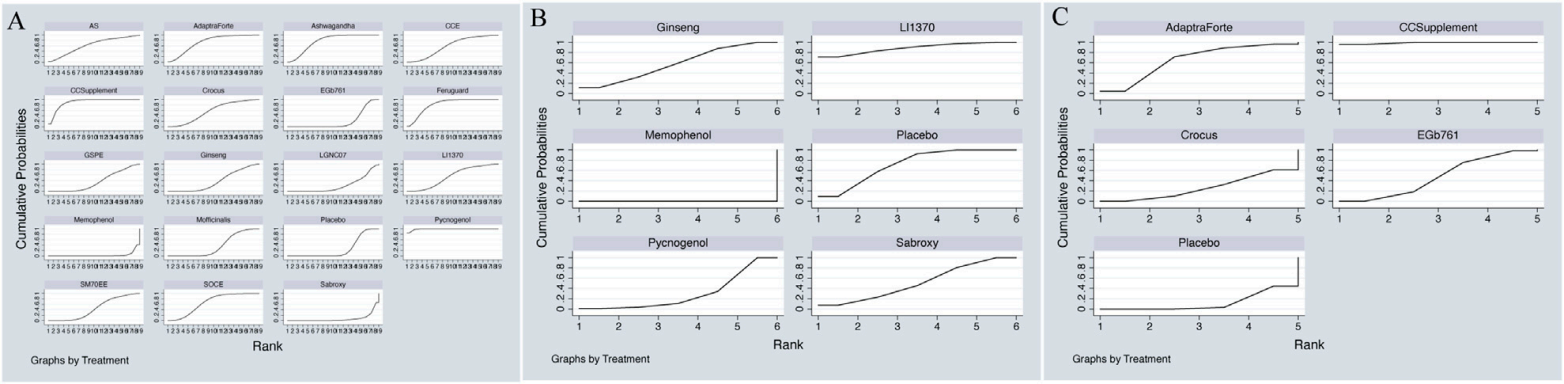
**(A)** SUCRA plot for cognitive function, **(B)** SUCRA plot for daily living, **(C)** SUCRA plot for psychological wellbeing.

The network meta-analysis of cognitive function revealed a clear hierarchy of effects among the 18 plant extracts.Pycnogenol demonstrated the largest and most statistically robust effect size against placebo (SMD = 0.70, 95% CI = 0.45, 0.95). These included CCSupplement (SMD = 0.26, 95% CI = [-0.52, 1.04]), Ferguar (SMD = 0.25, 95% CI = [-0.05, 0.55]), and Ashwagandha (SMD = 0.45, 95% CI = [0.14, 0.89]), all of which exhibited some effects, such as Memophenol (SMD = 0.23, 95% CI = [-0.05, 0.51]). Among the seven highly effective interventions, medium-to-large effect sizes were observed. This suggests the existence of several highly effective interventions. In contrast, some extracts showed no significant difference from placebo, highlighting the variability in efficacy across different plant-derived products. The probability ranking based on the SMD analysis showed Pycnogenol ranked first for improving cognitive function (SUCRA: 98.8%), as shown in Figure 5A. A comparison between the two interventions is presented in Table 2. To provide a more comprehensive overview of ranking probabilities, the SUCRA values for all interventions are presented in Table 3. The league table (Table 2) provides pairwise comparisons for all interventions. Notably, the direct comparison between the top-ranked intervention, Pycnogenol, and the second-ranked, CCSupplement, revealed a small, non-statistically significant difference in favor of Pycnogenol (SMD = 0.07; 95% CI [-0.18, 0.32]). However, Pycnogenol showed a statistically significant and large advantage over other interventions ranked lower, such as Hovenia (SMD = 1.00; 95% CI [0.75, 1.25]).

**TABLE 3:** SUCRA values for cognitive function based on SMD.

Intervention	SUCRA (%)	Intervention	SUCRA (%)
Pycnogenol	98.8	SM70EE	61.5
CCSupplement	91.5	Ginseng	59.1
Feruguard	85.2	Mofficinalis	57.0
Ashwagandha	80.4	GSPE	54.2
AdaptraForte	78.1	Placebo	50.0
SOCE	73.2	LGNC07	47.5
AS	70.9	EGb761	44.8
CCE	68.5	Sabroxy	42.1
Crocus	65.4	Memophenol	39.8
LI1370	62.1		

#### 3.4.2 Mild cognitive impairment of daily living

All P-values for indirect and direct comparisons across studies were tested for consistency and inconsistency, with most P-values greater than 0.05, indicating an acceptable level of consistency across studies. Details are provided in Supplementary Figure S2.

The results of the network meta-analysis indicated that, relative to the control group’s placebo, Pycnogenol [MD = 4.28, 95% CI = (3.51, 5.06)] and Memophenol [MD = 1.34, 95% CI = (0.94, 1.74)] were superior to the control group in improving daily living activities. The probability ranking of different plant extract interventions for effectiveness in improving daily living activities ranked Pycnogenol first in SUCRA (100.0%), as shown in Figure 5B. A comparison between the two interventions is presented in Table 4. The complete league table for this outcome is available in Supplementary Table S5. To further clarify the relative effectiveness, the SUCRA values of all included interventions for daily living activities are provided in Table 5. As shown in the league table (Table 4), the top-ranked intervention, Pycnogenol, demonstrated a statistically significant and large effect when compared to the second-ranked Memophenol (MD = 4.28, 95% CI = [3.51, 5.06]). This indicates a clear superiority of Pycnogenol for this outcome.

**TABLE 4:** League table on daily living.

Pycnogenol	LI1370	Placebo	Ginseng	Sabroxy	Memophenol
Pycnogenol	−4.03 (−5.02, −3.04)	−4.28 (−5.06, −3.51)	−4.38 (−5.27, −3.49)	−4.45 (−5.35, −3.54)	−5.62 (−6.50, −4.75)
4.03 (3.04,5.02)	LI1370	−0.26 (−0.87,0.36)	−0.36 (−1.11,0.39)	−0.42 (−1.19,0.35)	−1.60 (−2.33, −0.87)
4.28 (3.51,5.06)	0.26 (−0.36,0.87)	Placebo	−0.10 (−0.53,0.33)	−0.16 (−0.62,0.30)	−1.34 (−1.74, −0.94)
4.38 (3.49,5.27)	0.36 (−0.39,1.11)	0.10 (−0.33,0.53)	Ginseng	−0.07 (−0.70,0.57)	−1.24 (−1.83, −0.65)
4.45 (3.54,5.35)	0.42 (−0.35,1.19)	0.16 (−0.30,0.62)	0.07 (−0.57,0.70)	Sabroxy	−1.18 (−1.78, −0.57)
5.62 (4.75,6.50)	1.60 (0.87,2.33)	1.34 (0.94,1.74)	1.24 (0.65,1.83)	1.18 (0.57,1.78)	Memophenol

**TABLE 5:** SUCRA values for daily living.

Intervention	SUCRA (%)
Pycnogenol	100
Memophenol	85.7
LI1370	72.6
Ginseng	65.3
Sabroxy	58.4
Placebo	18.1

#### 3.4.3 Mild cognitive impairment of psychological wellbeing

All P-values for indirect and direct comparisons across studies were tested for consistency and inconsistency, and all P-values were greater than 0.05, indicating an acceptable level of consistency across studies. Details are provided in Supplementary Figure S3.

The results of the network meta-analysis indicated that CCSupplement [MD = 1.47, 95% CI = (0.82, 2.13)] was superior to the control group in improving mental and psychological scores relative to placebo. The probability ranking of plant extract interventions for improving mental and psychological scores showed CCSupplement ranked first in SUCRA (98.9%), as shown in Figure 5C. A comparison between the two interventions is presented in Table 6. The complete league table for psychological wellbeing is provided in Supplementary Table S6. To provide a more comprehensive overview of the ranking, the SUCRA values of all interventions for psychological wellbeing are presented in Table 7. The pairwise comparisons in Table 6, highlighting the findings for psychological wellbeing. The top-ranked CCSupplement showed a significant advantage over placebo (MD = −1.47, 95% CI = [-1.93, −1.02]). The direct comparison between CCSupplement and the second-ranked Adaptraforte indicated a non-statistically significant trend in favor of CCSupplement (MD = −0.26, 95% CI = [-1.14, 0.61]).

**TABLE 6:** League table on psychological wellbeing.

CCSupplement	AdaptraForte	EGb761	Crocus	Placeb
CCSupplement	−0.86 (−1.84,0.12)	−1.17 (−1.89, −0.44)	−1.39 (−2.32, −0.46)	−1.47 (−2.13, −0.82)
0.86 (−0.12,1.84)	AdaptraForte	−0.31 (−1.11,0.49)	−0.53 (−1.52,0.46)	−0.61 (−1.35,0.12)
1.17 (0.44,1.89)	0.31 (−0.49,1.11)	EGb761	−0.22 (−0.95,0.51)	−0.31 (−0.62,0.01)
1.39 (0.46,2.32)	0.53 (−0.46,1.52)	0.22 (−0.51,0.95)	Crocus	−0.09 (−0.75,0.58)
1.47 (0.82,2.13)	0.61 (−0.12,1.35)	0.31 (−0.01,0.62)	0.09 (−0.58,0.75)	Placebo

**TABLE 7:** SUCRA values psychological wellbeing.

Intervention	SUCRA (%)
CCSupplement	98.9
AdaptraForte	91.2
EGb761	83.3
Crocus	71.1
Placebo	55.7

### 3.5 Safety and tolerability

Of the 19 included trials, 15 reported on safety and tolerability. Overall, the investigated botanical drugs were well-tolerated. The incidence of adverse events in the intervention groups was generally low and comparable to that in the placebo groups. The most commonly reported side effects were mild and transient gastrointestinal complaints (e.g., nausea, stomach upset) and headaches. No serious adverse events directly attributable to the interventions were reported in any of the studies. Dropout rates due to adverse events were also similar between the intervention and placebo arms across the studies that reported this metric. However, the methods and detail of safety reporting varied considerably among the trials, with some providing only general statements about tolerability. A detailed summary of reported adverse events from each study is provided in Supplementary Table S8.

### 3.6 Publication bias test

**FIGURE 6:**
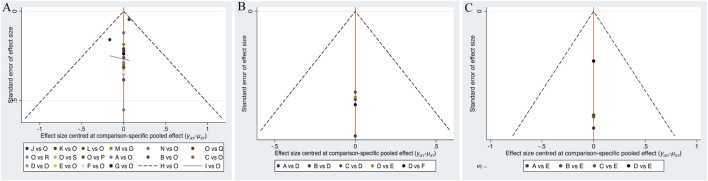
**(A)** Funnel plot on publication bias for cognitive function, **(B)** Funnel plot on publication bias for daily living, **(C)** Funnel plot on publication bias for psychological wellbeing.

Separate funnel plots were constructed for all outcome indicators to test for potential publication bias. Visual inspection of the funnel plots did not suggest significant publication bias (Wallace et al., 2009). Details are shown in Figures 6A–C.

## 4 Discussion

MCI most commonly presents with memory impairment symptoms that differ from normal aging, including frequent forgetfulness of daily events, difficulty recalling newly learned information, challenges in maintaining prolonged attention, reduced language expression, and a noticeable impact on daily life (Anand and Schoo, 2024). Although numerous drugs have been studied for MCI, no specific medication has been conclusively proven effective in its treatment.

Cognitive function screening tools, such as the Mini-Mental State Examination (MMSE), Montreal Cognitive Assessment (MoCA), and Alzheimer’s Disease Assessment Scale-cognitive subscale (ADAS-cog), effectively differentiate between normal cognition and MCI by assessing memory, attention, language, and executive function (Zhuang et al., 2021). Notably, MMSE and MoCA scores increase with better cognitive function, so a positive mean difference (MD) reflects improvement, whereas for ADAS-cog, which increases with greater impairment, a negative MD indicates improvement.

In recent years, several studies have explored the potential therapeutic effects of plant extracts on MCI, yet no research has evaluated which specific intervention is most effective. Thus, this study employed a Bayesian network meta-analysis to rank the effectiveness of 18 plant extract interventions, providing evidence-based guidance for selecting natural remedies for MCI patients.

This network meta-analysis, which synthesized data from 19 trials and 4,956 patients, provides a comprehensive synthesis of the currently available evidence on 18 plant extracts for MCI management. However, the findings must be interpreted with considerable caution in light of the methodological limitations identified across many of the primary studies, as will be discussed further. Our analysis identified Pycnogenol as the most effective intervention for improving both cognitive function, showing a large positive effect size (SMD = 0.85), and activities of daily living (ADL). This key finding warrants further discussion regarding the underlying mechanisms and clinical implications of Pycnogenol. A standardized extract from French maritime pine bark, Pycnogenol has been shown to significantly enhance working and spatial memory and positively affect psychomotor function (Belcaro et al., 2014). Animal studies confirm its neuroprotective effects via reducing oxidative stress, inflammatory factors, and regulating apoptosis (Xia et al., 2017). Since age-related oxidative stress impairs memory encoding pathways, antioxidants like Pycnogenol may counteract cognitive decline (Bastianetto and Quirion, 2004; Joseph et al., 2009).

Beyond Pycnogenol, our analysis also highlighted the significant cognitive-enhancing effects of other extracts, notably medium-to-large effect sizes were observed. These included CCSupplement (SMD = 0.26, 95% CI = [-0.52, 1.04]), Ferguar (SMD = 0.25, 95% CI = [-0.05, 0.55]), and Ashwagandha (SMD = 0.45, 95% CI = [0.14, 0.89]), all of which exhibited some effects, such as Memophenol (SMD = 0.23, 95% CI = [-0.05, 0.51]). Among the seven highly effective interventions, medium-to-large effect sizes were observed. This suggests the existence of several highly effective interventions. In contrast, some extracts showed no significant difference from placebo, highlighting the variability in efficacy across different plant-derived products.

Declines in cognitive function adversely impact patients’ quality of life, including self-care ability and independence in basic ADL, increasing the need for assistance and risk of injury (Hussenoeder et al., 2020). Consistent with its effects on cognition, our findings also highlighted Pycnogenol’s superiority in improving ADL compared to placebo, a result that aligns with prior research Pycnogenol’s antioxidant and anti-inflammatory properties reduce pro-inflammatory cytokines and β-amyloid plaques, contributing to therapeutic benefits in neurodegenerative diseases (Nattagh-Eshtivani et al., 2022; Paarmann et al., 2019). Enhanced memory and attention likely underlie the observed quality of life improvements.

Mental health is also a concern in MCI, with anxiety and depression commonly observed due to cognitive decline and social withdrawal (Orgeta et al., 2022). Tools like the Neuropsychiatric Inventory (NPI) and Geriatric Depression Scale (GDS) assess psychological symptoms comprehensively (Ginsberg et al., 2019). A key finding of our systematic review analysis was the superior performance of Cosmos caudatus Supplement (CCSupplement; Cosmos caudatus; commonly known as Ulam Raja or King’s Salad) in its beneficial role as a natural health supplement, attributable to its strong antioxidant and anti-inflammatory properties. Its potent effects on mental and oxidative stress are metabolites such as quercetin, and other flavonoids and phenolic acids. Chronic neuroinflammation and oxidative stress are well-established contributors not only to cognitive decline but also to mood disorders like depression and anxiety, which are highly prevalent in MCI patients. By mitigating these pathological processes in the brain, CCSupplement may help alleviate depressive symptoms and improve overall emotional stability. Furthermore, findings from the trial by study (You et al., 2021; You et al., 2020), which contributed data to our analysis, align with this hypothesis, demonstrating that supplementation led to significant improvements in mood and quality of life scores in the MCI population.

This meta-analysis observed heterogeneity, particularly in study regions and participant gender distributions, which may affect generalizability. Cultural, genetic, and environmental differences, including dietary patterns and baseline health—could influence botanical intervention efficacy. Gender imbalances may bias outcomes; thus, future research should target more homogeneous populations or perform pre-specified subgroup analyses to clarify these effects.

Furthermore, MCI itself is a heterogeneous clinical syndrome with various underlying etiologies (e.g., Alzheimer’s disease, vascular pathology). The therapeutic effects of plant extracts could vary significantly across these subtypes. Unfortunately, the primary studies included in our analysis generally did not stratify their participants or report outcomes based on MCI subtypes (e.g., amnestic vs. non-amnestic), which precluded any subgroup analysis to investigate this potential variation. This lack of detailed patient characterization in the source literature is a significant limitation of the current evidence base and highlights a critical direction for future research.

Additionally, the included studies used various cognitive, functional, and psychological scales with differing sensitivity and specificity, potentially impacting pooled SMD precision. Standardization of validated outcome measures in future MCI trials is crucial for reliable meta-analytic conclusions.

Not all studies assessed all three primary outcomes (cognition, daily living, psychological wellbeing) comprehensively, limiting full efficacy profiling for each botanical intervention. For example, the psychological effects of Pycnogenol remain unclear, underscoring the need for comprehensive outcome assessment in future research.

Recent studies suggest that CCSupplement may exert psychological benefits by activating Nrf2-mediated antioxidant pathways and inhibiting neuroinflammation, stabilizing mood and enhancing neuroprotection. CCSupplement and similar supplements synergistically improve mental health by modulating antioxidant, anti-inflammatory, and neuroprotective mechanisms, including NF-κB and JAK-STAT pathway inhibition and neurotrophic factor upregulation (Wu et al., 2025; Liao et al., 2020; Godos et al., 2020). Although direct human evidence is limited, these mechanisms are well-supported in the literature.

It is also valuable to consider conceptual subgroups based on the primary mechanisms of the most effective botanical drugs. Our findings suggest that successful interventions may fall into distinct mechanistic classes. For instance, Pycnogenol and GSPE represent a class of potent antioxidant and vasodilatory agents, whose benefits likely stem from reducing oxidative stress and improving cerebral blood flow. In contrast, Ashwagandha represents an “adaptogen” class, which is thought to enhance cognitive resilience by modulating the body’s stress response (e.g., regulating cortisol levels) in addition to its antioxidant effects. Meanwhile, extracts like LI1370 (Bacopa monnieri) are considered “nootropics” that may directly influence neurotransmitter systems involved in memory and learning. While the current evidence base is too sparse to conduct a formal statistical subgroup analysis by these categories, recognizing these distinct pharmacological profiles is essential for generating new hypotheses and designing future trials that could compare these different approaches.

An essential consideration for any therapeutic option is its safety and tolerability. Our review found that the botanical drugs included were generally reported as safe and well-tolerated, with most adverse events being mild and comparable in frequency to placebo. This favorable safety profile is a significant advantage, particularly for the MCI population, which is often elderly and may have multiple comorbidities. However, this finding must be interpreted with caution. The quality and comprehensiveness of safety reporting were inconsistent across the included trials. Many studies lacked systematic and active monitoring for adverse events. Therefore, while the current evidence is reassuring, a definitive conclusion on the long-term safety and potential for drug interactions for these botanical interventions cannot be drawn. Future trials must incorporate rigorous and standardized safety monitoring protocols.

Overall, our study has important clinical implications. Pycnogenol and CCSupplement significantly enhance cognitive function, activities of daily living, and psychosocial health in MCI patients. Physicians might consider incorporating these plant extracts as therapeutic options in clinical practice.

## 5 Strengths and limitations

The primary strength of this study lies in its comprehensive scope, synthesizing data from 19 trials and providing the first network meta-analysis to systematically compare a wide array of botanical extracts for Mild Cognitive Impairment (MCI). However, the conclusions are subject to several important limitations, primarily inherited from the included studies.

First, and most critically, the methodological quality of the primary evidence base is a major concern. As our risk of bias assessment revealed, a large proportion of studies presented an “unclear” risk of bias, particularly in the fundamental domains of allocation concealment and the blinding of participants and personnel. This poor or non-reporting is highly problematic, as it introduces a substantial potential for selection, performance, and detection biases. This uncertainty significantly tempers any strong conclusions about the “efficacy” of these preparations. While our analysis reflects the best available evidence, it is crucial to acknowledge that these methodological weaknesses mean the true effect sizes could be overestimated. The findings should therefore be viewed as preliminary and hypothesis-generating rather than definitive.

Second, the evidence base is characterized by significant heterogeneity. This includes not only variations in study regions and participant gender distributions but also the clinical heterogeneity within the MCI population itself (e.g., different underlying pathologies), which could influence treatment effects. A key limitation stemming from the source data is our inability to perform subgroup analyses by sex or population, as none of the included trials reported outcomes stratified in this manner. This lack of granular data in the primary literature prevents a deeper exploration of heterogeneity and underscores a critical gap in the current research landscape. We strongly urge future RCTs in this field to prespecify and report outcomes for these important subgroups.

Finally, other limitations include the variations in the sensitivity and specificity of the scales used for outcome assessment and the fact that not all studies assessed all three primary outcomes (cognition, daily living, and psychological wellbeing). These factors underscore the need for further, higher-quality research with standardized, comprehensive outcome assessments to address these limitations.

## 6 Conclusion

Based on our systematic review and network meta-analysis, we recommend Pycnogenol for improving cognitive function, where it demonstrated the largest and most consistent beneficial effect (SMD = 0.85), and for enhancing quality of daily life, and CCSupplement for enhancing mental psychology. Overall, although the studies on Pycnogenol did not include psychological assessments, it demonstrated more substantial effects in enhancing cognitive function and quality of daily life in patients with mild cognitive impairment, making it the most recommended plant extract.

## Data Availability

The original contributions presented in the study are included in the article/Supplementary Material, further inquiries can be directed to the corresponding authors.
