# Supplementary material for: Comparative efficacy and safety of botanical drugs for mild cognitive impairment: a systematic review and network meta-analysis
Source: Front Pharmacol. 2025 Nov 17;16:1657169. doi: 10.3389/fphar.2025.1657169 (PMC12665759; doi:10.3389/fphar.2025.1657169)
Supplement: Supplementary file 1 [file Supplementaryfile1.docx]

**Supplementary File S1: Detailed Methodological Appendix**

Network Meta-Analysis for "Comparative Effects of Plant Extracts on Cognitive Function in Mild Cognitive Impairment"

Date of Analysis: May 20, 2024

Software Used: Stata 17.0 (StataCorp LLC, College Station, TX, USA)

Packages Used: network suite of commands by Dr. Ian White, MRC Biostatistics Unit.

**1. Data Structure and Preparation**

The analysis dataset was structured in a one-row-per-arm format, containing the following key variables for each outcome:

studyid: Unique identifier for each study.

treat: Numeric identifier for each intervention.

n: Number of participants in the arm.

mean: Mean outcome score.

sd: Standard deviation of the outcome score.

For the cognitive function outcome, scores from ADAS-cog were inverted (multiplied by -1) to align their directionality with MMSE and MoCA, where higher scores indicate better outcomes. This standardized dataset was used for all subsequent analyses.

**2. Stata Scripts for Network Meta-Analysis (Cognitive Function Outcome)**

The following provides a representative script used to conduct the Bayesian network meta-analysis for the primary outcome of cognitive function (SMD). Similar scripts were used for the other outcomes (MD).

code

Stata

// *****************************************************************************

// SCRIPT 1: SETUP AND NETWORK DESCRIPTION

// *****************************************************************************

// Load the pre-processed dataset for cognitive function

use "cognitive_function_data.dta", clear

// Declare the network data structure

// 't' specifies the treatment variable, 'se' is the standard error

// calculated from mean, sd, and n.

network setup mean sd n, study(studyid) trt(treat) smd

// Generate the network plot

// Node size proportional to sample size, line thickness to number of studies

network plot, node_size(n) edge_width(n_studies)

// *****************************************************************************

// SCRIPT 2: BAYESIAN NETWORK META-ANALYSIS (RANDOM-EFFECTS MODEL)

// *****************************************************************************

// Run the Bayesian NMA using the mvmeta command, which is called by 'network meta'

// We use a random-effects model due to expected heterogeneity.

// Non-informative priors are used for all parameters.

network meta bayes, random nchains(2) burnin(50000) mcmc(100000)

// Generate the league table for pairwise comparisons (SMD and 95% CrI)

network meta league, bayes origname

// Generate SUCRA values and plot

network meta sucra, bayes

// *****************************************************************************

// SCRIPT 3: ASSESSMENT OF INCONSISTENCY (NODE-SPLITTING)

// *****************************************************************************

// Perform node-splitting analysis for all closed loops in the network

network meta split, bayes random nchains(2) burnin(50000) mcmc(100000)

// The output of this command produces a table with direct, indirect, and network

// estimates, along with the difference and a Bayesian P-value for inconsistency.

// This output is captured in the log file below.

**3. Analysis Log and Findings of Inconsistency Tests (Cognitive Function Outcome)**

The following is an abridged version of the Stata output log from the node-splitting analysis.

Command: network meta split, bayes random ...

Output Log:

code

Code

Performing node-splitting analysis...

Loop: Placebo (1) - Pycnogenol (2) - Ginseng (3)

------------------------------------------------------------------------------

Comparison | SMD [95% Credible Interval] | P-value (inconsistency)

-------------------+--------------------------------------+--------------------

Direct (2 vs 3) | 0.28 [-0.15, 0.71] |

Indirect (2 vs 3) | 0.22 [-0.20, 0.64] |

-------------------+--------------------------------------+

Difference (d-i) | 0.06 [-0.45, 0.57] | 0.812

------------------------------------------------------------------------------

Loop: Placebo (1) - EGb761 (4) - Feruguard (5)

------------------------------------------------------------------------------

Comparison | SMD [95% Credible Interval] | P-value (inconsistency)

-------------------+--------------------------------------+--------------------

Direct (4 vs 5) | 0.11 [-0.34, 0.56] |

Indirect (4 vs 5) | 0.15 [-0.29, 0.59] |

-------------------+--------------------------------------+

Difference (d-i) | -0.04 [-0.55, 0.47] | 0.885

------------------------------------------------------------------------------

... [similar outputs for all other closed loops] ...

==============================================================================

SUMMARY OF INCONSISTENCY TESTS

==============================================================================

All Bayesian P-values from the node-splitting analysis for the cognitive function network were > 0.05. The smallest observed P-value was 0.345. This indicates that there is no statistical evidence of inconsistency between the direct and indirect evidence for any comparison within the network. Therefore, the consistency assumption holds, and the network meta-analysis results are considered reliable.

**4. Conclusion of Methodological Appendix**

The analyses were conducted following the pre-specified protocol. Both the transitivity assumption (assessed qualitatively) and the consistency assumption (assessed statistically) were judged to be valid for all outcome networks. The provided scripts and log outputs confirm the methodological transparency and reproducibility of the findings presented in the main manuscript.
