# Supplementary material for: Comparative efficacy and safety of botanical drugs for mild cognitive impairment: a systematic review and network meta-analysis
Source: Front Pharmacol. 2025 Nov 17;16:1657169. doi: 10.3389/fphar.2025.1657169 (PMC12665759; doi:10.3389/fphar.2025.1657169)
Supplement: Supplementary file 2 [file Table1.docx]

TableS1. Botanical Drugs Included: Taxonomy and Preparation Methods

| Author | Year | Product Name | Latin Name | Authority | Family | Preparation Description | |
| --- | --- | --- | --- | --- | --- | --- | --- |
| Amieva | 2013 | EGb761 | Ginkgo biloba L. | Carl Linnaeus | Ginkgoaceae | Industrial process centers on ethanol extraction and multi-step purification; impurities removed by physical and chemical methods; concentrated and standardized. Must meet pharmacopoeia standards (total flavonoids 24%±1%, terpene lactones 6%±0.6%, ginkgolic acids <5 ppm). | |
| Choi | 2022 | SM70EE | Spirulina maxima | Spirulina | Oscillatoriales / Cyanobacteria | Extracted from Spirulina maxima with 70% ethanol to obtain SM70EE (70% ethanol extract). | |
| Choudhary | 2017 | Ashwagandha | Withania somnifera (L.) Dunal | Ashwagandha, Indian ginseng, Winter cherry | Solanaceae | Root or whole plant extracted via water or ethanol extraction to produce Ashwagandha extract or related preparations. | |
| Dimpfel | 2020 | AdaptraForte | Withania somnifera (L.) Dunal and Pfaffia paniculata Mart. ex DC. | Ashwagandha, Indian ginseng, Winter cherry and Suma, Brazilian ginseng | Solanaceae and Amaranthaceae | Roots of Ashwagandha and Brazilian ginseng extracted with water or ethanol, filtered and concentrated to yield the AdaptraForte composite extract. | |
| Gschwind | 2017 | LI1370 | Panax notoginseng (Burkill) F.H.Chen | Notoginseng, Tienchi, Sanchi | Araliaceae | Roots extracted with 70% ethanol, filtered, concentrated, and dried to obtain standardized LI1370 extract. | |
| Hosoi | 2018 | Pycnogenol | Pinus pinaster Aiton | Maritime pine, Cluster pine | Pinaceae | Crushed bark extracted with water or ethanol, filtered, concentrated, and dried to produce standardized maritime pine bark extract. | |
| Jung | 2021 | SOCE | - |  | - | - |  |
| Lopresti | 2021 | Sabroxy | Oroxylum indicum (L.) Kurz | Indian trumpet tree | Bignoniaceae | Dried bark of Indian trumpet tree extracted with methanol, filtered, concentrated, and dried; rich in flavonoids such as Oroxylin A, Baicalein, and Chrysin. | |
| Lopresti | 2023 | Memophenol | Vitis vinifera L. and Vaccinium angustifolium Aiton | Grape and Lowbush blueberry | Vitaceae and Ericaceae | Grape seed and lowbush blueberry extracted with ethanol or water, concentrated, and standardized to form the polyphenol complex Memophenol. | |
| Moeko | 2023 | Mofficinalis | Melissa officinalis L. | Lemon balm | Lamiaceae | Leaves extracted with ethanol or water, filtered, concentrated, and dried to obtain Mofficinalis extract. | |
| Park | 2019 | Ginseng | Panax ginseng C.A. Mey. | Asian ginseng | Araliaceae | Roots extracted with ethanol or water, concentrated, and dried to prepare ginseng extract. | |
| Park | 2011 | LGNC07 | Panax ginseng C.A. Mey. | Asian ginseng | Araliaceae | Ginseng roots extracted with 70% ethanol, filtered, concentrated, and dried to yield standardized LGNC07 extract. | |
| Robinson | 2020 | CCE | Camellia sinensis (L.) Kuntze | Tea plant | Theaceae | New leaves extracted by reflux with water or ethanol, filtered, and concentrated to prepare tea polyphenol extract (CCE). | |
| Magda | 2016 | Crocus | Crocus sativus L. | Saffron | Iridaceae | Saffron stigmas crushed and extracted with ethanol or water, concentrated, and dried to obtain Crocus extract. | |
| You | 2021 | CCSupplement | Ophiocordyceps sinensis (formerly Cordyceps sinensis) | Chinese caterpillar fungus | Ophiocordycipitaceae | Fruiting bodies or fermented mycelia extracted with ethanol or water, concentrated, and dried to prepare CCSupplement. | |
| Gavrilova | 2014 | EGb761 | Ginkgo biloba L. | Carl Linnaeus | Ginkgoaceae | Industrial process centers on ethanol extraction and multi-step purification; impurities removed by physical and chemical methods; concentrated and standardized. Must meet pharmacopoeia standards (total flavonoids 24%±1%, terpene lactones 6%±0.6%, ginkgolic acids <5 ppm). | |
| Kudoh | 2020 | Feruguard | Ferula asafoetida L. | Asafoetida | Apiaceae | Resin extracted with ethanol or water, filtered, concentrated, and dried to obtain Feruguard asafoetida extract. | |
| Ito | 2018 | AS | Withania somnifera (L.) Dunal | Ashwagandha, Indian ginseng, Winter cherry | Solanaceae | Roots extracted with water or ethanol, concentrated, and dried to prepare Ashwagandha (AS) extract. | |
| Li | 2023 | GSPE | Vitis vinifera L. | Grape | Vitaceae | Grape seeds extracted with ethanol or water, filtered, concentrated, and spray-dried to obtain GSPE (grape seed proanthocyanidin extract). | |
