# Supplementary material for: Comparative efficacy and safety of botanical drugs for mild cognitive impairment: a systematic review and network meta-analysis
Source: Front Pharmacol. 2025 Nov 17;16:1657169. doi: 10.3389/fphar.2025.1657169 (PMC12665759; doi:10.3389/fphar.2025.1657169)
Supplement: Supplementary file 3 [file Table8.docx]

### Supplementary Table S8: Summary of Reported Adverse Events and Tolerability

| **Study (First Author, Year)** | **Intervention** | **Reported Adverse Events (AEs) in Intervention Group** | **Reported AEs in Placebo Group** | **Conclusion on Tolerability (as stated by authors)** |
| --- | --- | --- | --- | --- |
| Amieva, 2013 | EGb761 | Rate of AEs (e.g., headache, GI upset) not significantly different from placebo over the long-term follow-up. | Similar rate of AEs reported. | Good tolerability profile. |
| Choi, 2022 | SM70EE | Mild gastrointestinal (GI) discomfort (n=2), transient headache (n=1). | Mild GI discomfort (n=1). | Considered safe and well-tolerated. |
| Choudhary, 2017 | Ashwagandha | No significant adverse events were reported by the participants. | No significant adverse events were reported by the participants. | Excellent safety and tolerability. |
| Dimpfel, 2020 | AdaptraForte | Safety data not explicitly reported in the manuscript. | Safety data not explicitly reported in the manuscript. | Tolerability not explicitly stated. |
| Gavrilova, 2014 | EGb761 | Bleeding events (n=3), GI symptoms (n=5). Rates were not statistically different from placebo. | Bleeding events (n=2), GI symptoms (n=4). | The preparation was safe and well-tolerated. |
| Gschwind, 2017 | LI1370 | Mild nausea (n=2), dry mouth (n=1). | Mild nausea (n=1). | Well-tolerated with minor side effects. |
| Hosoi, 2018 | Pycnogenol® | Minor skin rash (n=1), which resolved without intervention. | Headache (n=1). | Very well-tolerated. |
| Ito, 2018 | AS | Safety data not explicitly reported. | Safety data not explicitly reported. | Tolerability not explicitly stated. |
| Jung, 2021 | SOCE | GI upset (n=3). | GI upset (n=2), dizziness (n=1). | No significant difference in AE rates; well-tolerated. |
| Kudoh, 2020 | Feruguard | Mild headache (n=2). | Mild headache (n=3). | Considered safe. |
| Li, 2023 | GSPE | No adverse events were reported. | No adverse events were reported. | High safety and tolerability. |
| Lopresti, 2021 | Sabroxy® | Nausea (n=3), transient dizziness (n=2). | Nausea (n=2). | Generally well-tolerated. |
| Lopresti, 2023 | Memophenol™ | No significant difference in the incidence of AEs compared to placebo. | Similar AE profile. | Well-tolerated. |
| Noguchi-Shinohara, 2023 | Mofficinalis | Abdominal discomfort (n=1). | No AEs reported. | Good tolerability. |
| Park, K. C., 2019 | Ginseng | Mild insomnia (n=2). | Mild headache (n=1). | Considered safe with few side effects. |
| Park, S. K., 2011 | LGNC07 | Safety data not reported in detail; mentioned as "no serious AEs". | Mentioned as "no serious AEs". | Assumed to be well-tolerated. |
| Robinson, 2020 | CCE | No AEs were reported during the study period. | No AEs were reported during the study period. | High safety profile. |
| Tsolaki, 2016 | Crocus | Nausea and decreased appetite reported in 2 participants. | Dry mouth reported in 1 participant. | Good tolerability. |
| You, 2021 | CCSupplement | No adverse events were reported by any participants. | No adverse events were reported by any participants. | Excellent tolerability and safety. |
