# Supplementary material for: Comparative efficacy and safety of botanical drugs for mild cognitive impairment: a systematic review and network meta-analysis
Source: Front Pharmacol. 2025 Nov 17;16:1657169. doi: 10.3389/fphar.2025.1657169 (PMC12665759; doi:10.3389/fphar.2025.1657169)
Supplement: Supplementary file 6 [file Table3.docx]

Supplementary Table S3: Botanical Drugs Included: Taxonomy, Preparation, and Standardization Details

| **Intervention Name** | **Full Botanical Name (with Authority)** | **Family** | **Pharmacopeial Drug Name (if applicable)** | **Plant Part Used** | **Preparation and Standardization Details (as reported)** |
| --- | --- | --- | --- | --- | --- |
| EGb761 | *Ginkgo biloba* L. | Ginkgoaceae | *Ginkgo folium* | Leaf | Patented dry extract; standardized to 24% ginkgo flavone glycosides and 6% terpene lactones. |
| SM70EE | *Morus alba* L. | Moraceae | *Mori folium* | Leaf | Standardized ethanol extract; specific markers not reported. |
| Ashwagandha | *Withania somnifera* (L.) Dunal | Solanaceae | *Withaniae radix* | Root | High-concentration, full-spectrum root extract; standardized to ≥5% withanolides. |
| AdaptraForte | *Rhodiola rosea* L. / *Eleutherococcus senticosus* (Rupr. & Maxim.) Maxim. / *Schisandra chinensis* (Turcz.) Baill. | Crassulaceae / Araliaceae / Schisandraceae | *Rhodiolae radix* / *Eleutherococci radix* | Root / Root / Berry | Proprietary blend; specific ratios and standardization not reported. |
| LI1370 | *Bacopa monnieri* (L.) Wettst. | Plantaginaceae | *Brahmi* | Whole Plant | Proprietary standardized extract; standardized for bacosides content. |
| Pycnogenol® | *Pinus pinaster* Aiton | Pinaceae |  | Bark | Patented extract from French maritime pine bark; standardized to 65-75% procyanidins. |
| SOCE | *Sophora flavescens* Aiton / *Boswellia sacra* Flueck. | Fabaceae / Burseraceae | *Sophorae flavescentis radix* / *Olibanum* | Fruit / Resin | Complex extract; extraction method and standardization not fully described. |
| Sabroxy® | *Oroxylum indicum* (L.) Kurz | Bignoniaceae |  | Bark | Proprietary extract; standardized to ≥10% oroxylin A. |
| Memophenol™ | *Vitis vinifera* L. / *Vaccinium angustifolium* Aiton | Vitaceae / Ericaceae |  | Fruit / Fruit | Proprietary blend of extracts; standardized for flavonoid content. |
| Mofficinalis | *Melissa officinalis* L. | Lamiaceae | *Melissae folium* | Leaf | Standardized extract; standardized for rosmarinic acid content. |
| Ginseng | *Panax ginseng* C.A.Mey. | Araliaceae | *Ginseng radix rubra* | Root | Fermented red ginseng extract; standardized for ginsenoside content. |
| LGNC07 | *Angelica gigas* Nakai / *Cnidium officinale* Makino / *Gardenia jasminoides* J.Ellis | Apiaceae / Apiaceae / Rubiaceae |  | Root / Rhizome / Fruit | Complex herbal formula extract; specific standardization markers not reported. |
| CCE | *Cistanche tubulosa* (Schenk) Wight | Orobanchaceae | *Cistanches herba* | Stem | Extract standardized for phenylethanoid glycosides (echinacoside, acteoside). |
| Crocus | *Crocus sativus* L. | Iridaceae | *Croci stigma* | Stigma | Extract standardized for safranal and crocin content. |
| CCSupplement | *Cosmos caudatus* Kunth | Asteraceae |  | Leaf | Standardized aqueous extract; standardized for total phenolic and flavonoid content. |
| AS | *Angelica archangelica* L. | Apiaceae | *Angelicae archangelicae radix* | Root | Extract of angelica root; specific standardization details not provided. |
| GSPE | *Vitis vinifera* L. | Vitaceae |  | Seed | Grape Seed Proanthocyanidin Extract; standardized for proanthocyanidin content. |
| Feruguard | Ferulic acid & *Angelica archangelica* L. | Synthetic & Apiaceae |  | Synthetic & Root | Combination product; ferulic acid is synthetic; angelica extract standardization not detailed. |
