# Supplementary material for: Comparative efficacy and safety of botanical drugs for mild cognitive impairment: a systematic review and network meta-analysis
Source: Front Pharmacol. 2025 Nov 17;16:1657169. doi: 10.3389/fphar.2025.1657169 (PMC12665759; doi:10.3389/fphar.2025.1657169)
Supplement: Supplementary file 8 [file Supplementaryfile2.docx]

Search strategy on all searched databases.

PubMed：

| #1 | “Cognitive Dysfunction” [MeSH Terms] |
| --- | --- |
| #2 | (((((((((((((((((((((((((Cognitive Dysfunction[Title/Abstract]) OR (Cognitive Dysfunctions[Title/Abstract])) OR (Dysfunction, Cognitive[Title/Abstract])) OR (Dysfunctions, Cognitive[Title/Abstract])) OR (Cognitive Impairments[Title/Abstract])) OR (Cognitive Impairment[Title/Abstract])) OR (Impairment, Cognitive[Title/Abstract])) OR (Impairments, Cognitive[Title/Abstract])) OR (Cognitive Disorder[Title/Abstract])) OR (Cognitive Disorders[Title/Abstract])) OR (Disorder, Cognitive[Title/Abstract])) OR (Disorders, Cognitive[Title/Abstract])) OR (Mild Cognitive Impairment[Title/Abstract])) OR (Cognitive Impairment, Mild[Title/Abstract])) OR (Cognitive Impairments, Mild[Title/Abstract])) OR (Impairment, Mild Cognitive[Title/Abstract])) OR (Impairments, Mild Cognitive[Title/Abstract])) OR (Mild Cognitive Impairments[Title/Abstract])) OR (Cognitive Decline[Title/Abstract])) OR (Cognitive Declines[Title/Abstract])) OR (Decline, Cognitive[Title/Abstract])) OR (Declines, Cognitive[Title/Abstract])) OR (Mental Deterioration[Title/Abstract])) OR (Deterioration, Mental[Title/Abstract])) OR (Deteriorations, Mental[Title/Abstract])) OR (Mental Deteriorations[Title/Abstract]) |
| #3 | #1 OR #2 |
| #4 | “Plant Extracts” [MeSH Terms] |
| #5 | (((((Plant Extracts [Title/Abstract]) OR (Extracts, Plant[Title/Abstract])) OR (Plant Extract[Title/Abstract])) OR (Extract, Plant[Title/Abstract])) OR (Herbal Medicines[Title/Abstract])) OR (Medicines, Herbal[Title/Abstract]) |
| #6 | #4 OR #5 |
| #7 | #3 AND #6 |

Embase：

| Search | **Embase** |
| --- | --- |
| **#1** | 'cognitive dysfunction':ti,ab,kw OR 'cognitive dysfunctions':ti,ab,kw OR 'dysfunction, cognitive':ti,ab,kw OR 'dysfunctions, cognitive':ti,ab,kw OR 'cognitive impairments':ti,ab,kw OR 'cognitive impairment':ti,ab,kw OR 'impairment, cognitive':ti,ab,kw OR 'impairments, cognitive':ti,ab,kw OR 'cognitive disorder':ti,ab,kw OR 'cognitive disorders':ti,ab,kw OR 'disorder, cognitive':ti,ab,kw OR 'disorders, cognitive':ti,ab,kw OR 'mild cognitive impairment':ti,ab,kw OR 'cognitive impairment, mild':ti,ab,kw OR 'cognitive impairments, mild':ti,ab,kw OR 'impairment, mild cognitive':ti,ab,kw OR 'impairments, mild cognitive':ti,ab,kw OR 'mild cognitive impairments':ti,ab,kw OR 'cognitive decline':ti,ab,kw OR 'cognitive declines':ti,ab,kw OR 'decline, cognitive':ti,ab,kw OR 'declines, cognitive':ti,ab,kw OR 'mental deterioration':ti,ab,kw OR 'deterioration, mental':ti,ab,kw OR 'deteriorations, mental':ti,ab,kw OR 'mental deteriorations':ti,ab,kw |
| **#2** | 'plant extracts':ti,ab,kw OR 'extracts, plant':ti,ab,kw OR 'plant extract':ti,ab,kw OR 'extract, plant':ti,ab,kw OR 'herbal medicines':ti,ab,kw OR 'medicines, herbal':ti,ab,kw |
| #3 | #1 AND #2 |

Cochrane：

| Search | **Cochrane** |
| --- | --- |
| #1 | (Cognitive Dysfunction):ti,ab,kw OR (Cognitive Dysfunctions):ti,ab,kw OR (Dysfunction, Cognitive):ti,ab,kw OR (Dysfunctions, Cognitive):ti,ab,kw OR (Cognitive Impairments):ti,ab,kw |
| #2 | Cognitive Impairment):ti,ab,kw OR (Impairment, Cognitive):ti,ab,kw OR (Impairments, Cognitive):ti,ab,kw OR (Cognitive Disorder):ti,ab,kw OR (Cognitive Disorders):ti,ab,kw |
| #3 | (Disorder, Cognitive):ti,ab,kw OR (Disorders, Cognitive):ti,ab,kw OR (Mild Cognitive Impairment):ti,ab,kw OR (Cognitive Impairment, Mild):ti,ab,kw OR (Cognitive Impairments, Mild):ti,ab,kw |
| #4 | (Impairment, Mild Cognitive):ti,ab,kw OR (Impairments, Mild Cognitive):ti,ab,kw OR (Mild Cognitive Impairments):ti,ab,kw OR (Cognitive Decline):ti,ab,kw OR (Cognitive Declines):ti,ab,kw |
| #5 | (Decline, Cognitive):ti,ab,kw OR (Declines, Cognitive):ti,ab,kw OR (Mental Deterioration):ti,ab,kw OR (Deterioration, Mental):ti,ab,kw OR (Deteriorations, Mental):ti,ab,kw |
| #6 | (Cognitive Dysfunction):ti,ab,kw OR (Mental Deteriorations):ti,ab,kw |
| #7 | #1 or #2 or #3 or #4 or #5 or #6 |
| #8 | (Plant Extracts):ti,ab,kw OR (Extracts, Plant):ti,ab,kw OR (Plant Extract):ti,ab,kw OR (Extract, Plant):ti,ab,kw OR (Herbal Medicines):ti,ab,kw |
| #9 | (Plant Extracts):ti,ab,kw OR (Medicines, Herbal):ti,ab,kw |
| #10 | #8 or #9 |
| #11 | #7 and #10 |

Web of Science：

| Search | **Web of Science** |
| --- | --- |
| #1 | Cognitive Dysfunction (All Fields) or Cognitive Dysfunctions (All Fields) or Dysfunction, Cognitive (All Fields) or Dysfunctions, Cognitive (All Fields) or Cognitive Impairments (All Fields) or Cognitive Impairment (All Fields) or Impairment, Cognitive (All Fields) or Impairments, Cognitive (All Fields) or Cognitive Disorder (All Fields) or Cognitive Disorders (All Fields) or Disorder, Cognitive (All Fields) or Disorders, Cognitive (All Fields) or Mild Cognitive Impairment (All Fields) or Cognitive Impairment, Mild (All Fields) or Cognitive Impairments, Mild (All Fields) or Impairment, Mild Cognitive (All Fields) or Impairments, Mild Cognitive (All Fields) or Mild Cognitive Impairments (All Fields) or Cognitive Decline (All Fields) or Cognitive Declines (All Fields) or Decline, Cognitive (All Fields) or Declines, Cognitive (All Fields) or Mental Deterioration (All Fields) or Deterioration, Mental (All Fields) or Deteriorations, Mental (All Fields) or Mental Deteriorations (All Fields) |
| #2 | Plant Extracts (All Fields) or Extracts, Plant (All Fields) or Plant Extract (All Fields) or Extract, Plant (All Fields) or Herbal Medicines (All Fields) or Medicines, Herbal (All Fields) |
| #3 | **#1 AND #2** |
