# Supplementary material for: Comparative efficacy and safety of botanical drugs for mild cognitive impairment: a systematic review and network meta-analysis
Source: Front Pharmacol. 2025 Nov 17;16:1657169. doi: 10.3389/fphar.2025.1657169 (PMC12665759; doi:10.3389/fphar.2025.1657169)

The measures of statistical heterogeneity are presented for the psychological well-being in patients with MCI.


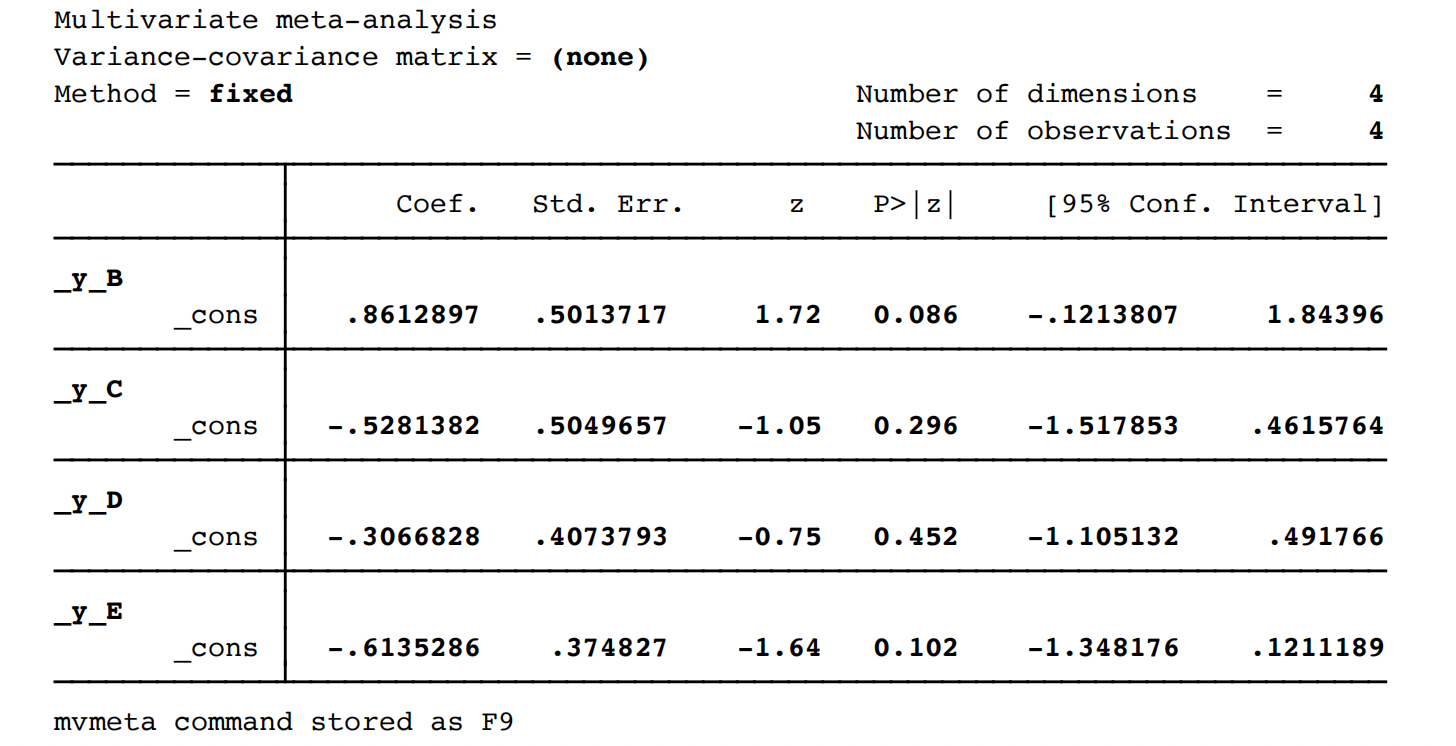

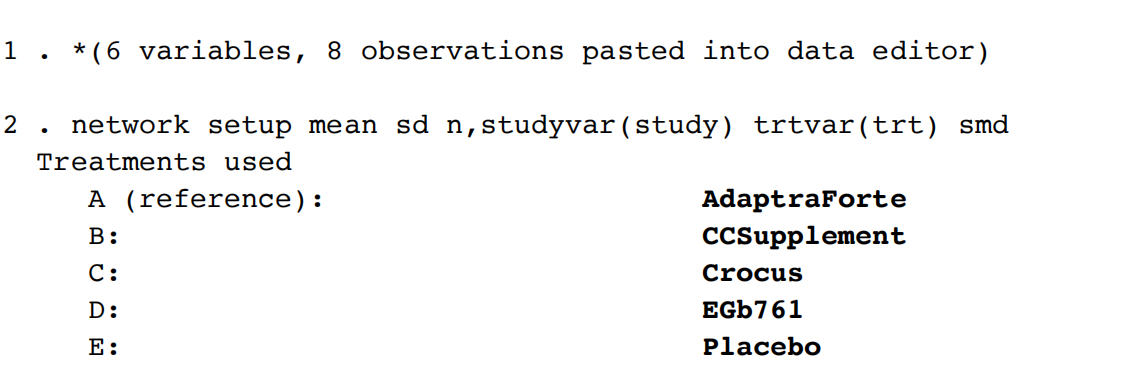

Supplement: Supplementary file 9 [file Table6.docx]
