# Supplementary material for: Comparative efficacy and safety of botanical drugs for mild cognitive impairment: a systematic review and network meta-analysis
Source: Front Pharmacol. 2025 Nov 17;16:1657169. doi: 10.3389/fphar.2025.1657169 (PMC12665759; doi:10.3389/fphar.2025.1657169)
Supplement: Supplementary file 10 [file DataSheet1.docx]

**Supplementary reference list**

[1] H. Amieva, C. Meillon, C. Helmer, P. Barberger-Gateau, J.F. Dartigues, Ginkgo biloba extract and long-term cognitive decline: a 20-year follow-up population-based study, PLoS One 8 (2013) e52755. 10.1371/journal.pone.0052755.

[2] W.Y. Choi, W.K. Lee, T.H. Kim, Y.K. Ryu, A. Park, Y.J. Lee, S.J. Heo, C. Oh, Y.C. Chung, D.H. Kang, The Effects of Spirulina maxima Extract on Memory Improvement in Those with Mild Cognitive Impairment: A Randomized, Double-Blind, Placebo-Controlled Clinical Trial, Nutrients 14 (2022). 10.3390/nu14183714.

[3] D. Choudhary, S. Bhattacharyya, S. Bose, Efficacy and Safety of Ashwagandha (Withania somnifera (L.) Dunal) Root Extract in Improving Memory and Cognitive Functions, J Diet Suppl 14 (2017) 599-612. 10.1080/19390211.2017.1284970.

[4] W. Dimpfel, L. Schombert, I.K. Keplinger-Dimpfel, A. Panossian, Effects of an Adaptogenic Extract on Electrical Activity of the Brain in Elderly Subjects with Mild Cognitive Impairment: A Randomized, Double-Blind, Placebo-Controlled, Two-Armed Cross-Over Study, Pharmaceuticals (Basel) 13 (2020). 10.3390/ph13030045.

[5] S.I. Gavrilova, U.W. Preuss, J.W. Wong, R. Hoerr, R. Kaschel, N. Bachinskaya, Efficacy and safety of Ginkgo biloba extract EGb 761 in mild cognitive impairment with neuropsychiatric symptoms: a randomized, placebo-controlled, double-blind, multi-center trial, Int J Geriatr Psychiatry 29 (2014) 1087-1095. 10.1002/gps.4103.

[6] Y.J. Gschwind, S.A. Bridenbaugh, S. Reinhard, U. Granacher, A.U. Monsch, R.W. Kressig, Ginkgo biloba special extract LI 1370 improves dual-task walking in patients with MCI: a randomised, double-blind, placebo-controlled exploratory study, Aging Clin Exp Res 29 (2017) 609-619. 10.1007/s40520-016-0699-y.

[7] M. Hosoi, G. Belcaro, A. Saggino, R. Luzzi, M. Dugall, B. Feragalli, Pycnogenol® supplementation in minimal cognitive dysfunction, J Neurosurg Sci 62 (2018) 279-284. 10.23736/s0390-5616.18.04382-5.

[8] N. Ito, H. Saito, S. Seki, F. Ueda, T. Asada, Effects of Composite Supplement Containing Astaxanthin and Sesamin on Cognitive Functions in People with Mild Cognitive Impairment: A Randomized, Double-Blind, Placebo-Controlled Trial, J Alzheimers Dis 62 (2018) 1767-1775. 10.3233/jad-170969.

[9] S.J. Jung, E.S. Jung, K.C. Ha, H.I. Baek, Y.K. Park, S.K. Han, S.W. Chae, S.O. Lee, Y.C. Chung, Efficacy and Safety of Sesame Oil Cake Extract on Memory Function Improvement: A 12-Week, Randomized, Double-Blind, Placebo-Controlled Pilot Study, Nutrients 13 (2021). 10.3390/nu13082606.

[10] C. Kudoh, T. Hori, S. Yasaki, R. Ubagai, T. Tabira, Effects of Ferulic Acid and Angelica archangelica Extract (Feru-guard( ®)) on Mild Cognitive Impairment: A Multicenter, Randomized, Double-Blind, Placebo-Controlled Prospective Trial, J Alzheimers Dis Rep 4 (2020) 393-398. 10.3233/adr-200211.

[11] B. Li, J. Cheng, G. Cheng, H. Zhu, B. Liu, Y. Yang, Q. Dai, W. Li, W. Bao, S. Rong, The effect of grape seed procyanidins extract on cognitive function in elderly people with mild cognitive impairment: A randomized, double-blind, placebo-controlled clinical trial, Heliyon 9 (2023) e16994. 10.1016/j.heliyon.2023.e16994.

[12] A.L. Lopresti, S.J. Smith, M. Majeed, P.D. Drummond, Effects of an Oroxylum indicum Extract (Sabroxy(®)) on Cognitive Function in Adults With Self-reported Mild Cognitive Impairment: A Randomized, Double-Blind, Placebo-Controlled Study, Front Aging Neurosci 13 (2021) 728360. 10.3389/fnagi.2021.728360.

[13] A.L. Lopresti, S.J. Smith, C. Pouchieu, L. Pourtau, D. Gaudout, V. Pallet, P.D. Drummond, Effects of a polyphenol-rich grape and blueberry extract (Memophenol™) on cognitive function in older adults with mild cognitive impairment: A randomized, double-blind, placebo-controlled study, Front Psychol 14 (2023) 1144231. 10.3389/fpsyg.2023.1144231.

[14] M. Noguchi-Shinohara, T. Hamaguchi, K. Sakai, J. Komatsu, K. Iwasa, M. Horimoto, H. Nakamura, M. Yamada, K. Ono, Effects of Melissa officinalis Extract Containing Rosmarinic Acid on Cognition in Older Adults Without Dementia: A Randomized Controlled Trial, J Alzheimers Dis 91 (2023) 805-814. 10.3233/jad-220953.

[15] S.K. Park, I.C. Jung, W.K. Lee, Y.S. Lee, H.K. Park, H.J. Go, K. Kim, N.K. Lim, J.T. Hong, S.Y. Ly, S.S. Rho, A combination of green tea extract and l-theanine improves memory and attention in subjects with mild cognitive impairment: a double-blind placebo-controlled study, J Med Food 14 (2011) 334-343. 10.1089/jmf.2009.1374.

[16] K.C. Park, H. Jin, R. Zheng, S. Kim, S.E. Lee, B.H. Kim, S.V. Yim, Cognition enhancing effect of panax ginseng in Korean volunteers with mild cognitive impairment: a randomized, double-blind, placebo-controlled clinical trial, Transl Clin Pharmacol 27 (2019) 92-97. 10.12793/tcp.2019.27.3.92.

[17] J.L. Robinson, J.M. Hunter, T. Reyes-Izquierdo, R. Argumedo, J. Brizuela-Bastien, R. Keller, Z.J. Pietrzkowski, Cognitive short- and long-term effects of coffee cherry extract in older adults with mild cognitive decline, Neuropsychol Dev Cogn B Aging Neuropsychol Cogn 27 (2020) 918-934. 10.1080/13825585.2019.1702622.

[18] M. Tsolaki, E. Karathanasi, I. Lazarou, K. Dovas, E. Verykouki, A. Karacostas, K. Georgiadis, A. Tsolaki, K. Adam, I. Kompatsiaris, Z. Sinakos, Efficacy and Safety of Crocus sativus L. in Patients with Mild Cognitive Impairment: One Year Single-Blind Randomized, with Parallel Groups, Clinical Trial, J Alzheimers Dis 54 (2016) 129-133. 10.3233/jad-160304.

[19] Y.X. You, S. Shahar, N.F. Rajab, H. Haron, H.M. Yahya, M. Mohamad, N.C. Din, M.Y. Maskat, Effects of 12 Weeks Cosmos caudatus Supplement among Older Adults with Mild Cognitive Impairment: A Randomized, Double-Blind and Placebo-Controlled Trial, Nutrients 13 (2021). 10.3390/nu13020434.
